# Supplementary material for: Organic Salts Based on Isoniazid Drug: Synthesis, Bioavailability and Cytotoxicity Studies
Source: Pharmaceutics. 2020 Oct 10;12(10):952. doi: 10.3390/pharmaceutics12100952 (PMC7600673; doi:10.3390/pharmaceutics12100952)
Supplement: Supplementary file 1 [file pharmaceutics-12-00952-s001.pdf]

pharmaceutics

MDPI

Supplementary Materials: Organic Salts Based on Isoniazid Drug: Synthesis, Bioavailability and Cytotoxicity Studies

Filipa Santos, Luís C. Branco and Ana Rita C. Duarte

Appendix A@NMR Spectra

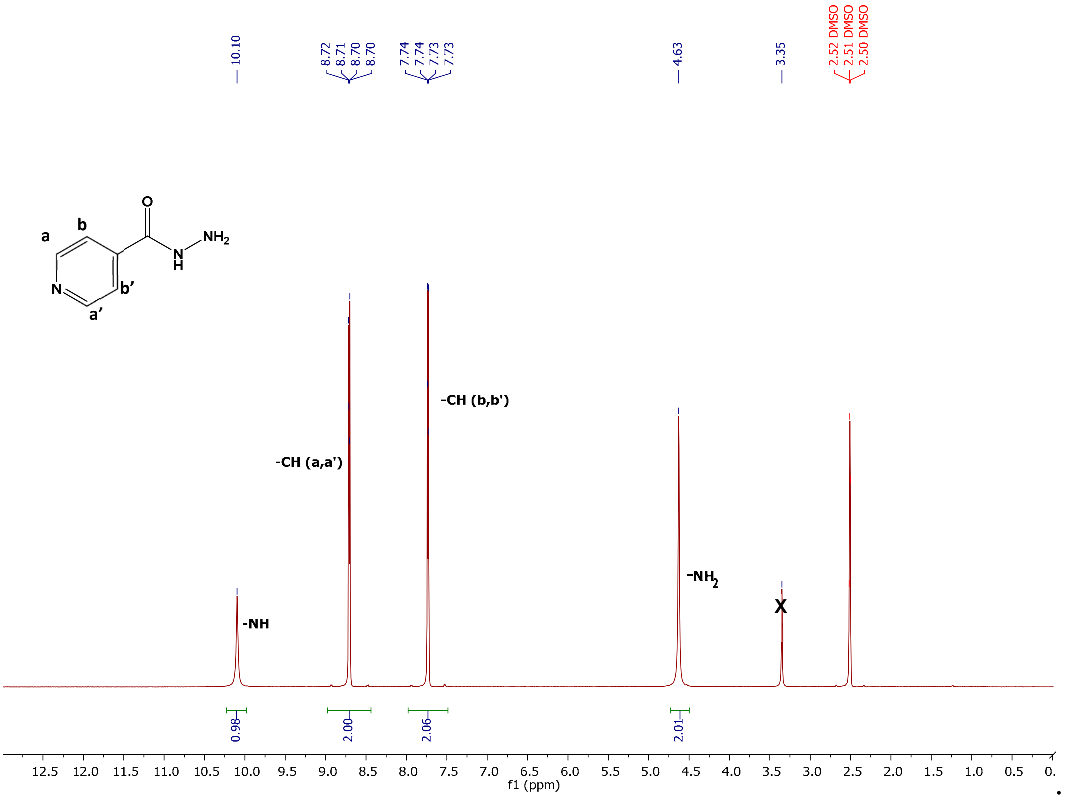

Figure S1. ^{1}H NMR spectra of INH.

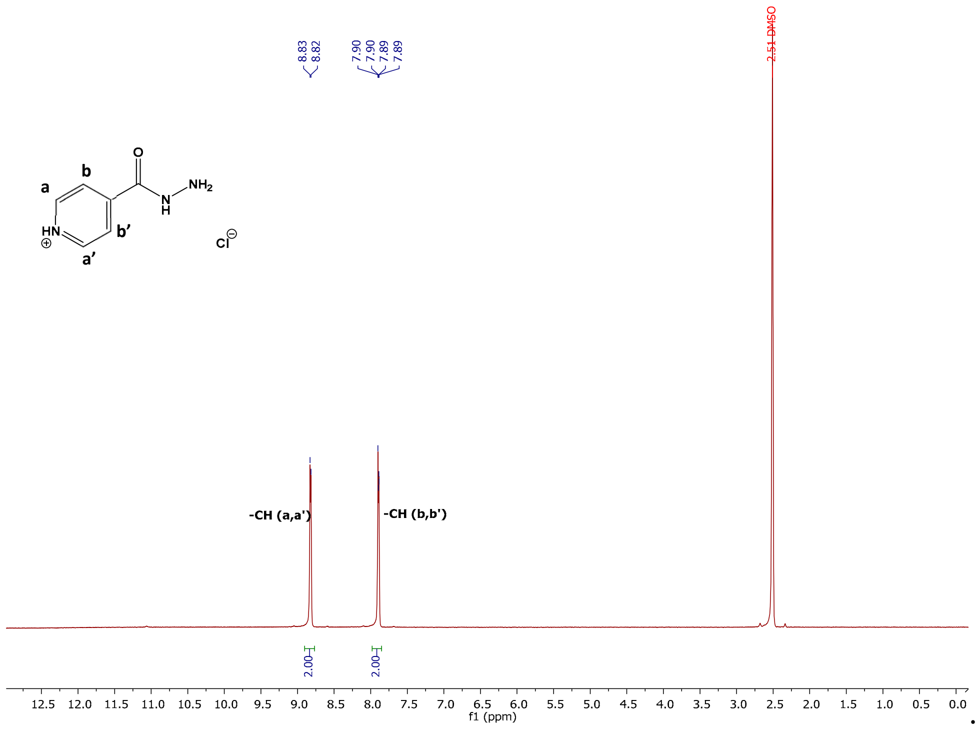

Figure S2. ^{1}H NMR spectra of [INH][Cl].

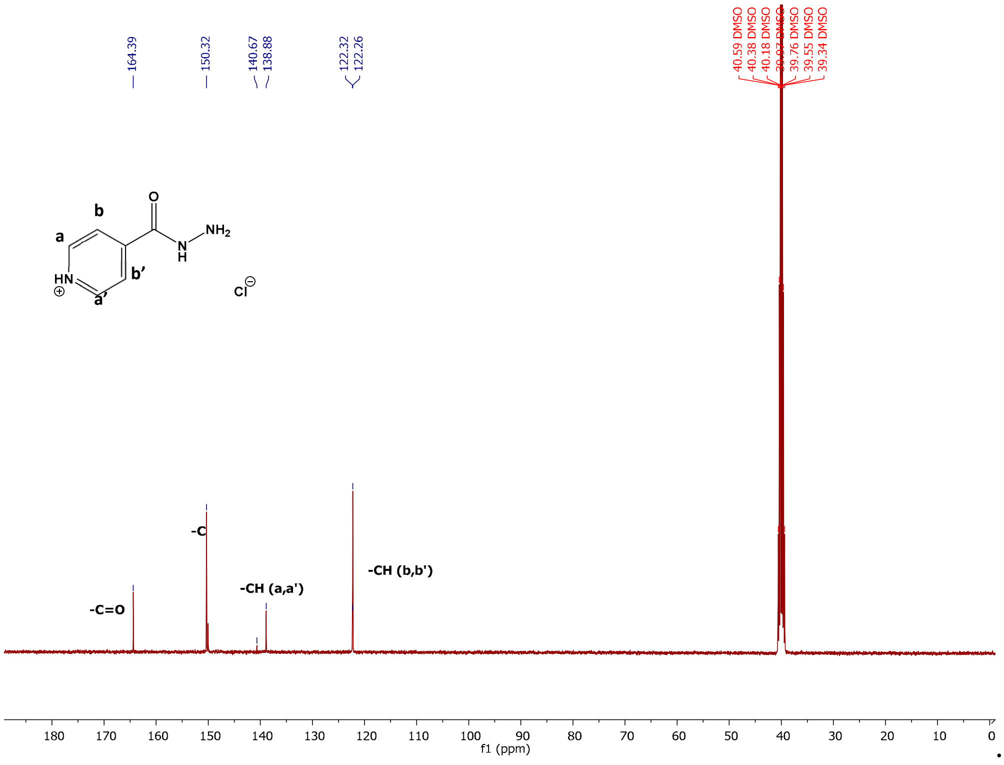

Figure S3. ^{13}C NMR spectra of [INH][Cl].

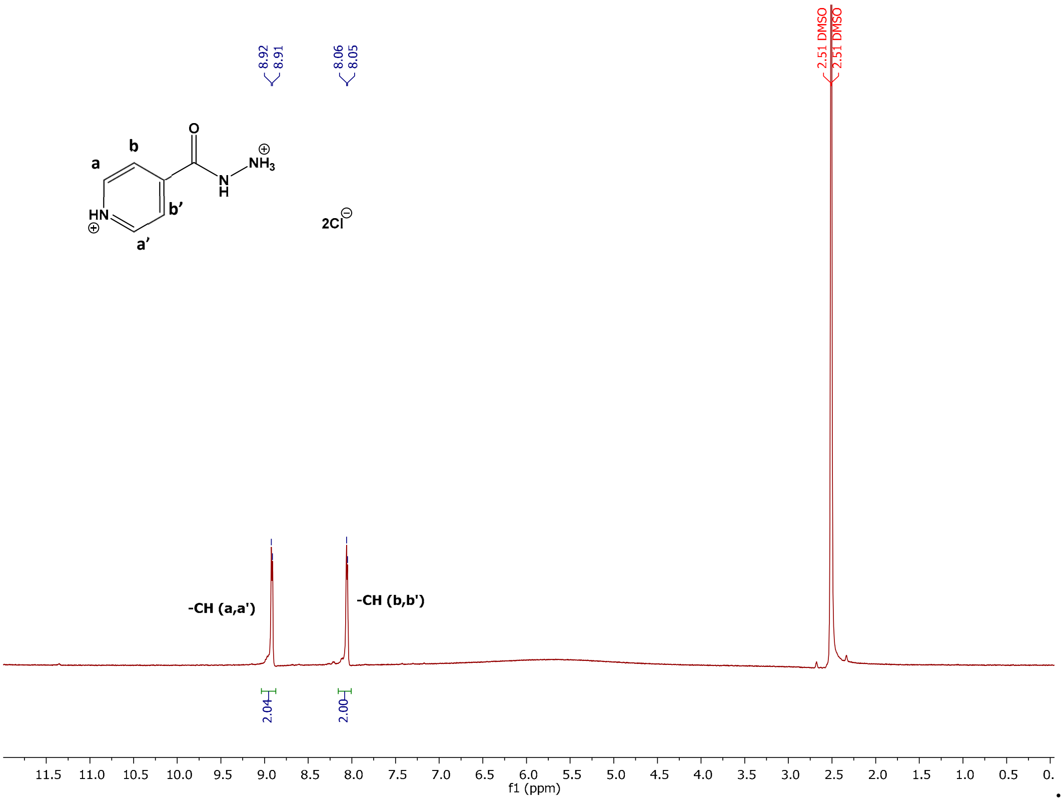

Figure S4. ^{1}H NMR spectra of [INH][Cl]$_2$.

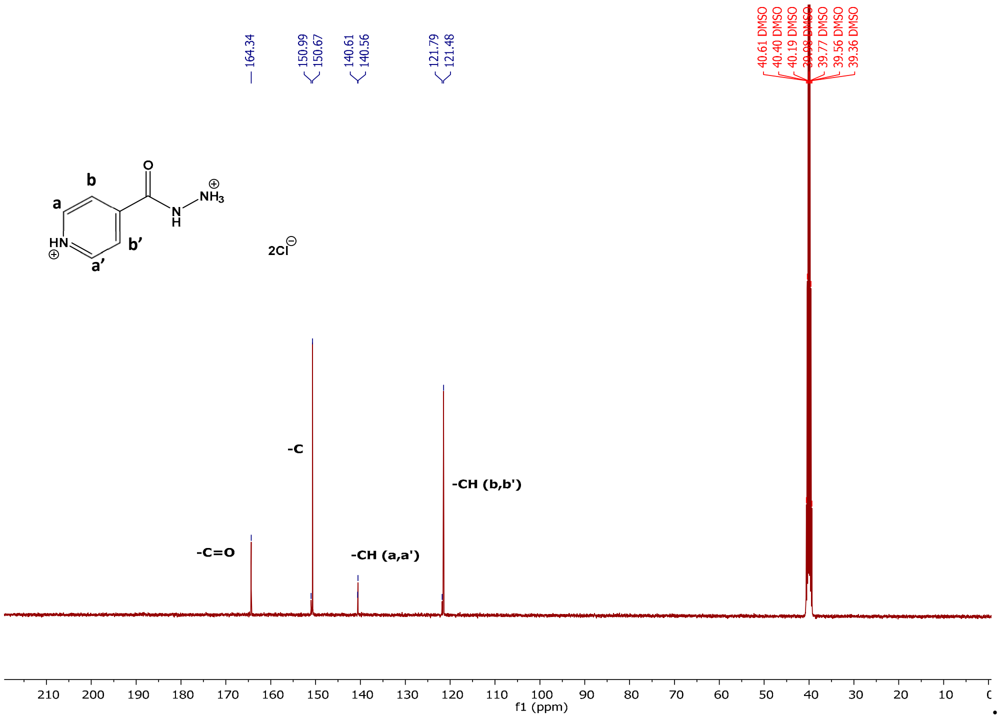

Figure S5. ^{13}C NMR spectra of [INH][Cl]$_2$.

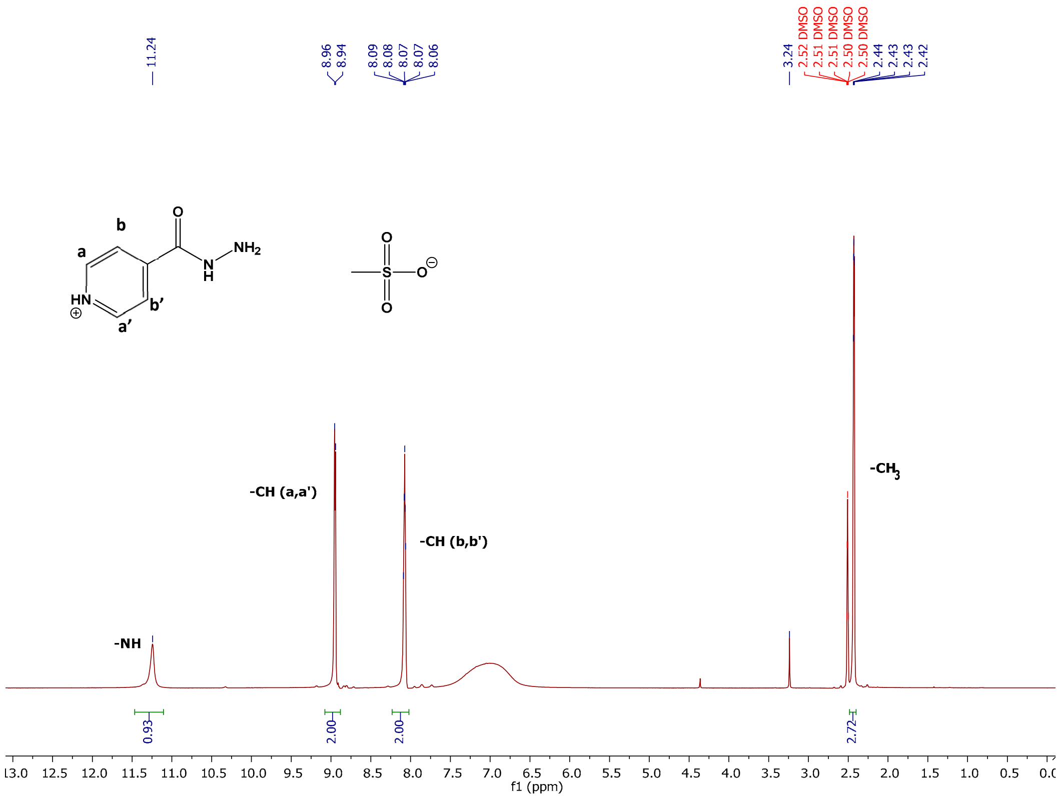

Figure S6. ¹H NMR spectra of [INH][MsO].

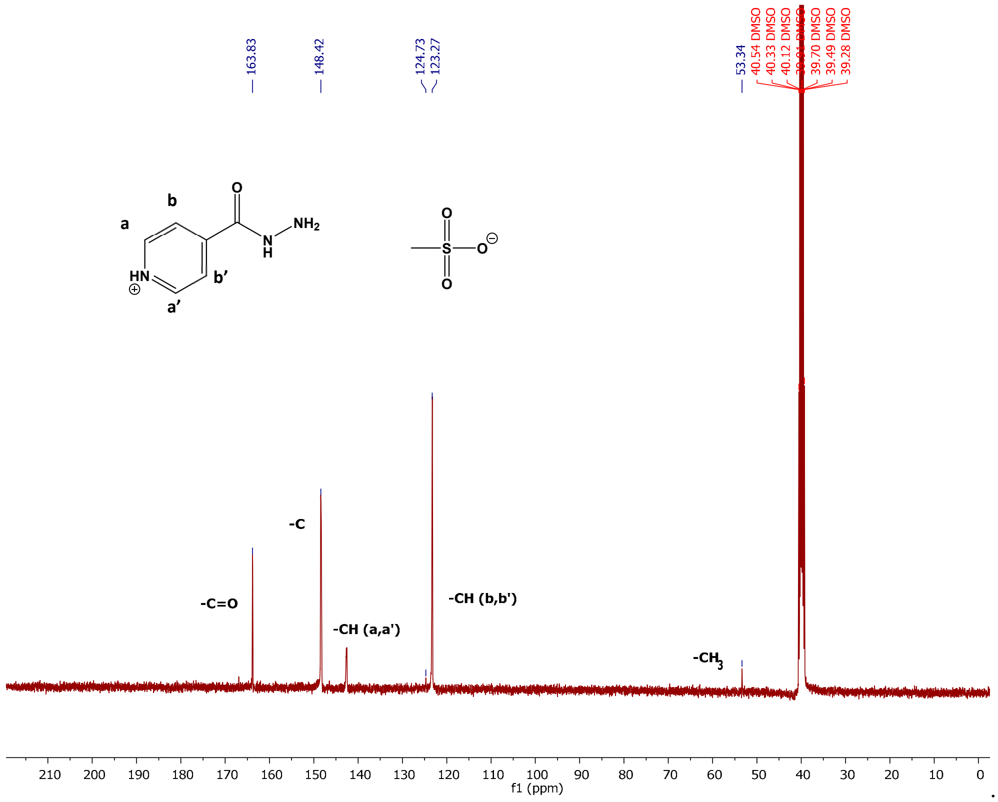

Figure S7. ¹³C NMR spectra of [INH][MsO].

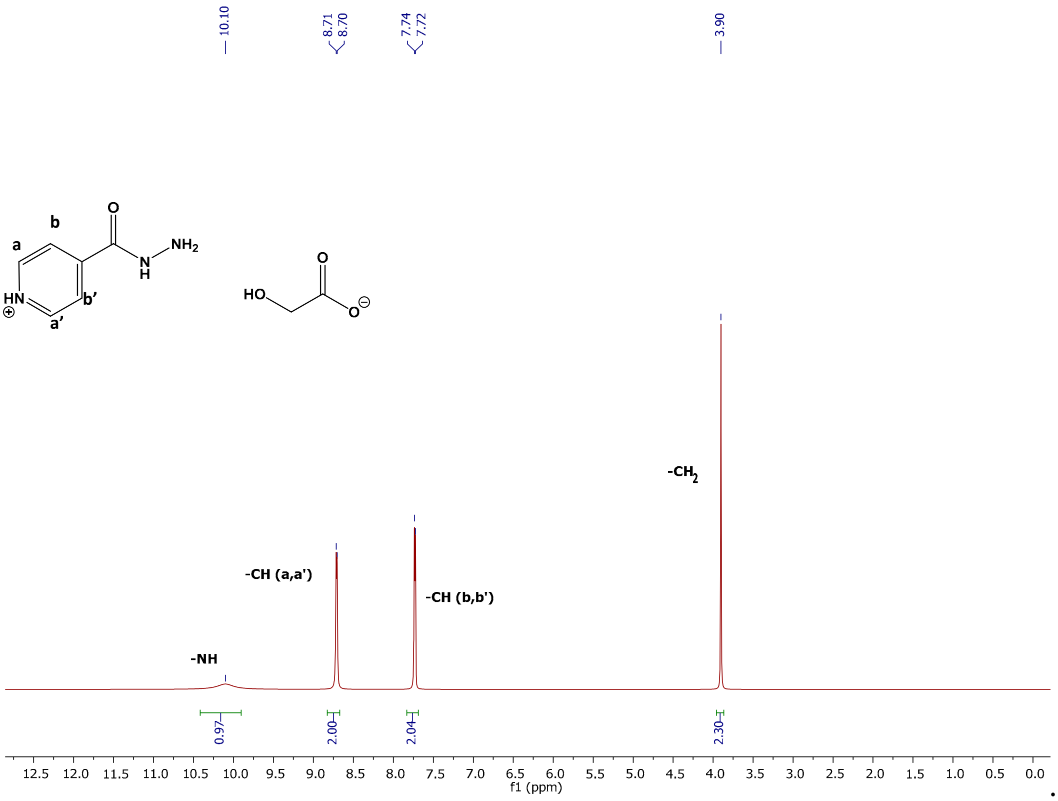

Figure S8. ^{1}H NMR spectra of [INH][GcO].

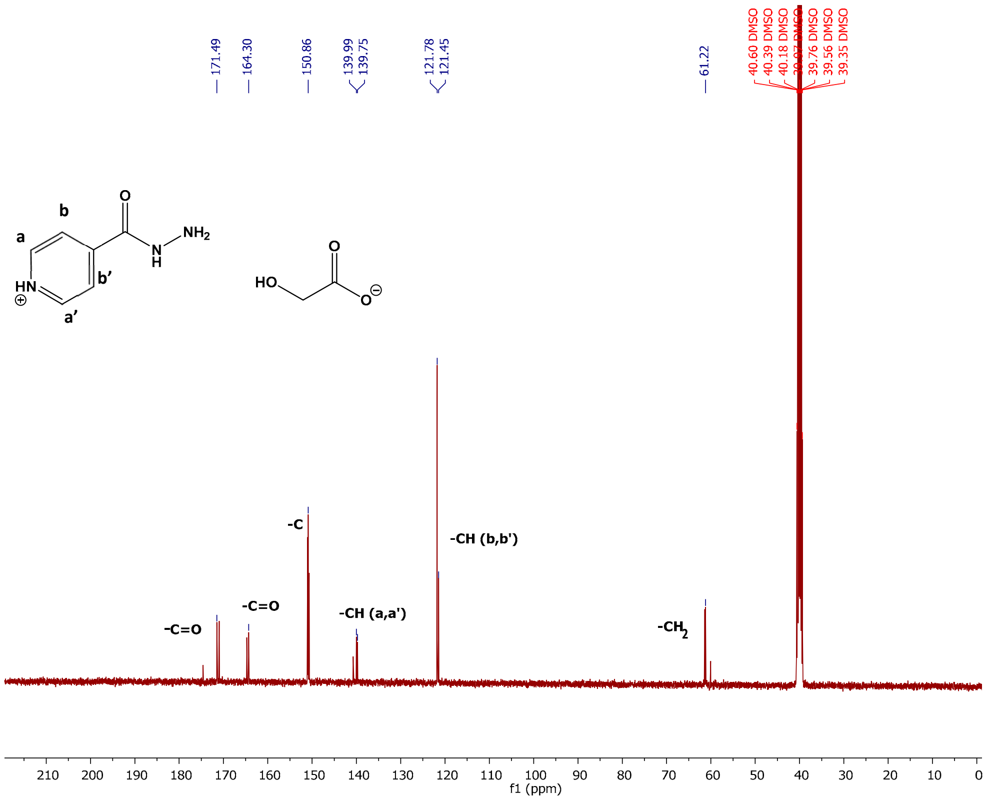

Figure S9. ^{13}C NMR spectra of [INH][GcO].

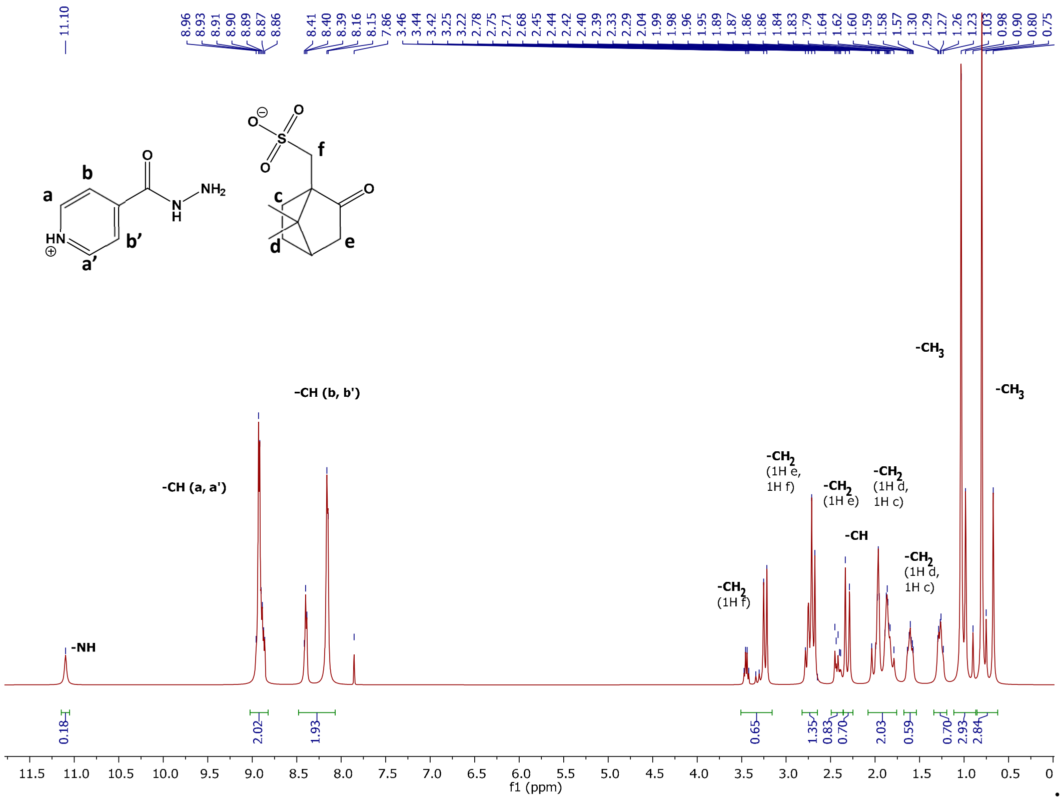

Figure S10. ^{1}H NMR spectra of [INH][S-CsO].

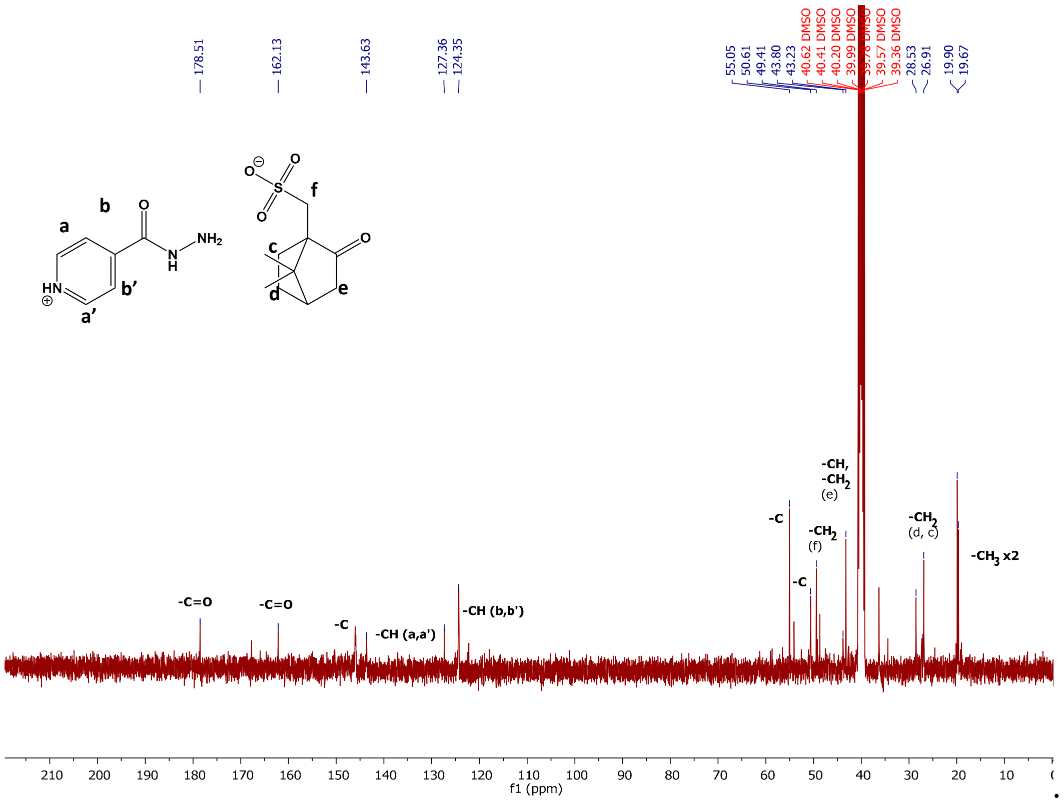

Figure S11. ^{13}C NMR spectra of [INH][S-CsO].

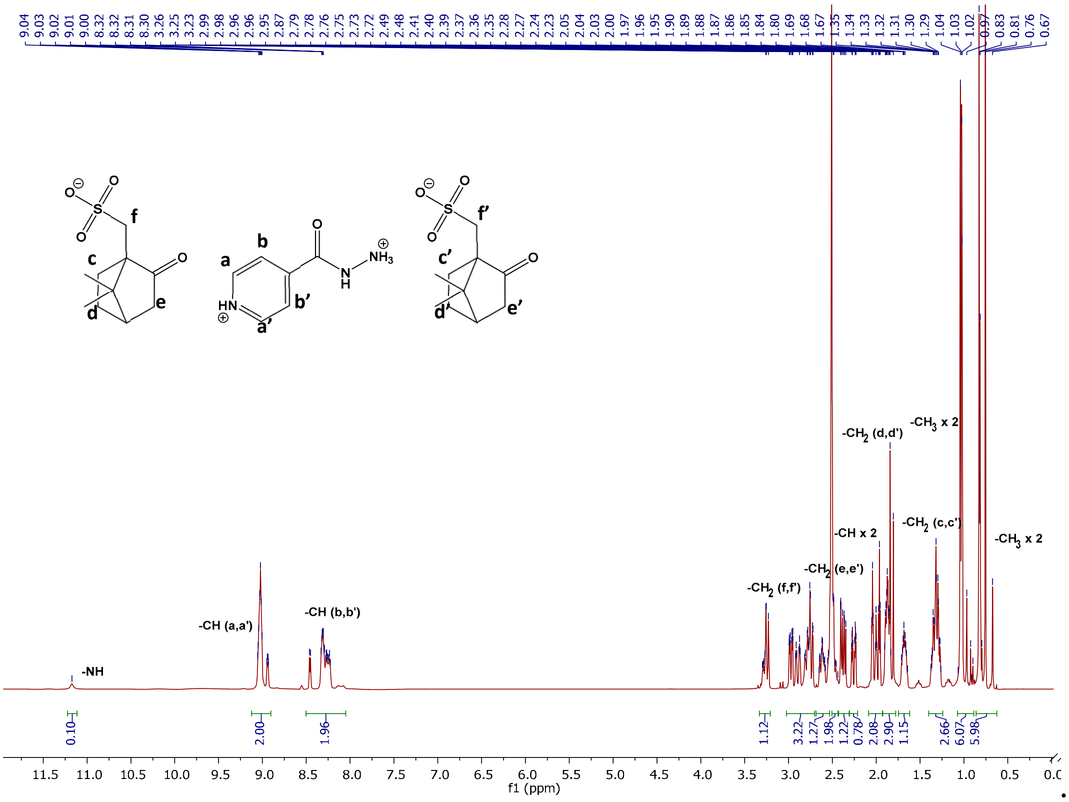

Figure S12. ^{1}H NMR spectra of [INH][S-CsO]2.

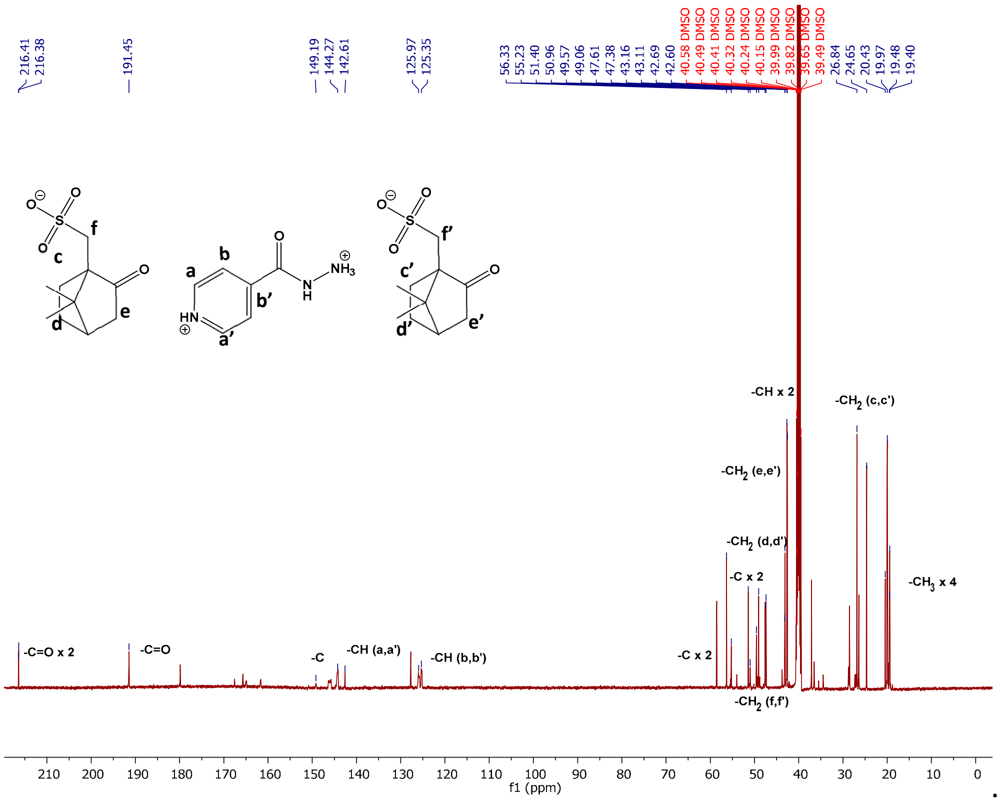

Figure S13. ^{13}C NMR spectra of [INH][S-CsO]2.

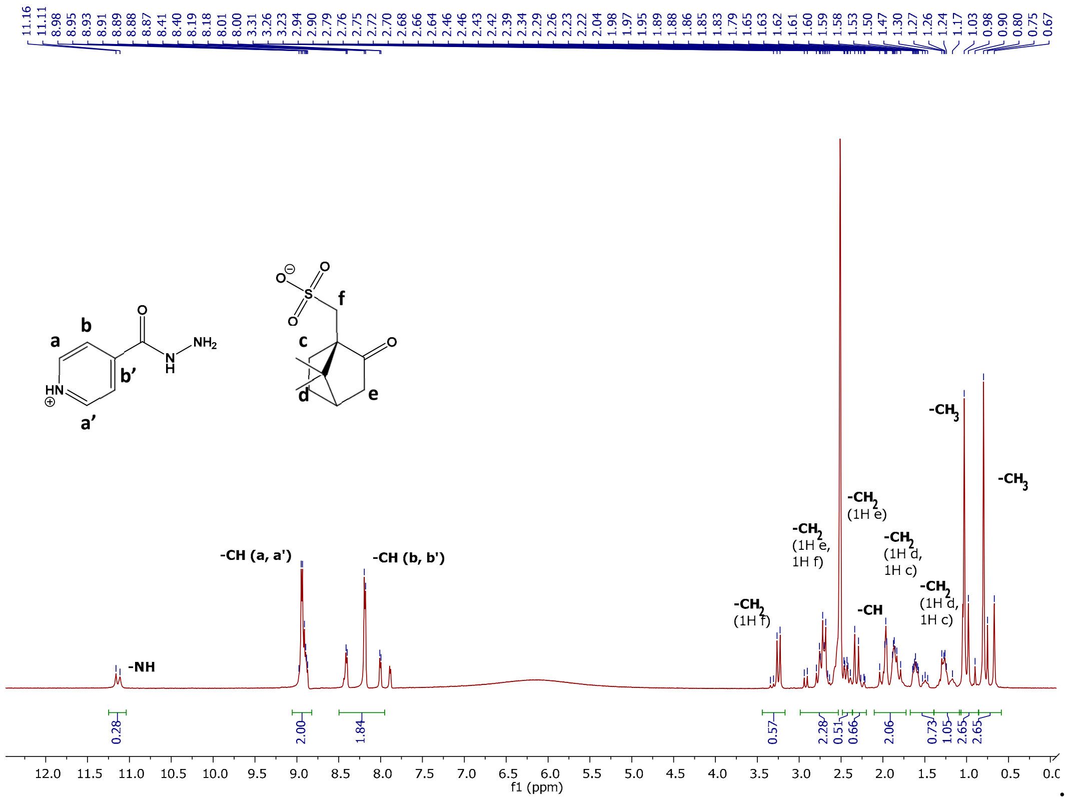

Figure S14. ^{1}H NMR spectra of [INH][R-CsO].

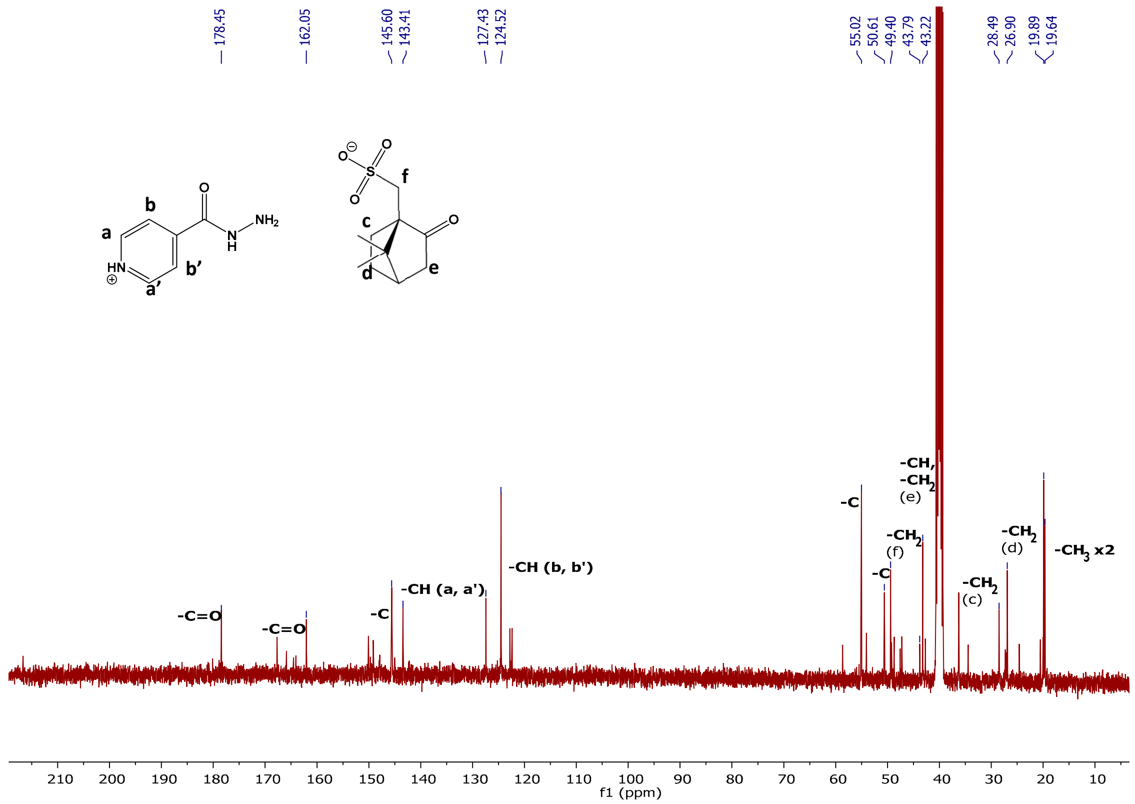

Figure S15. ^{13}C NMR spectra of [INH][R-CsO].

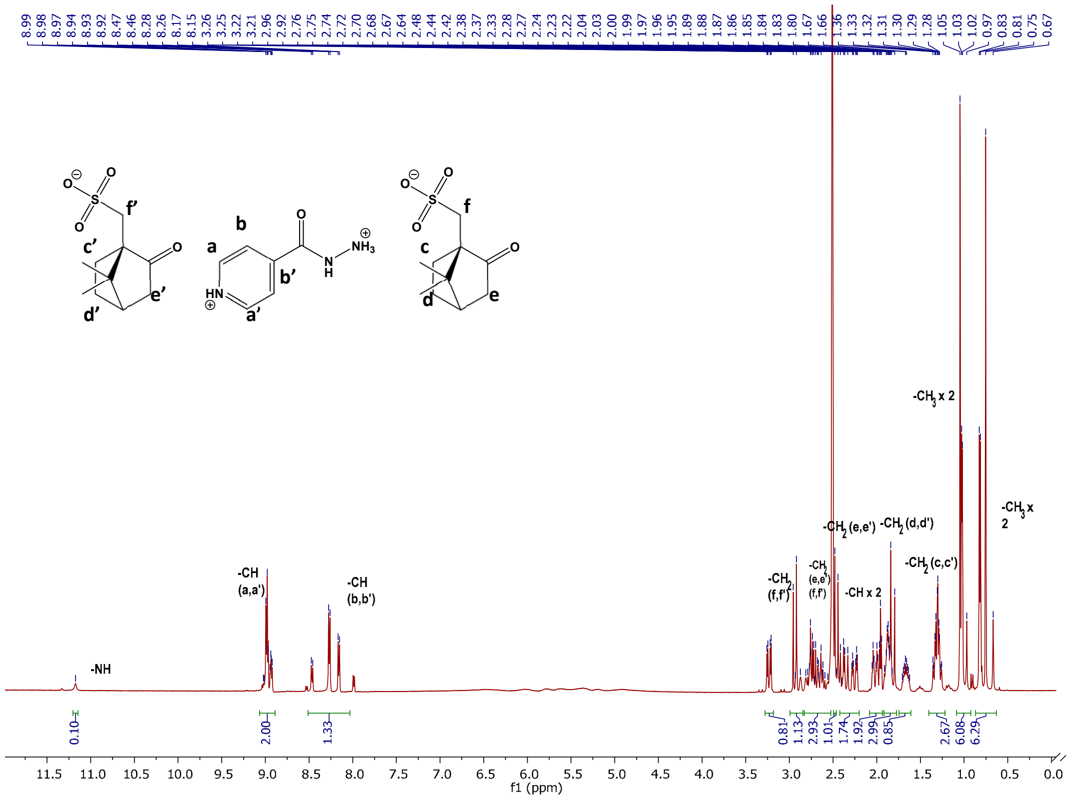

Figure S16. ^{1}H NMR spectra of [INH][R-CsO]$_2$.

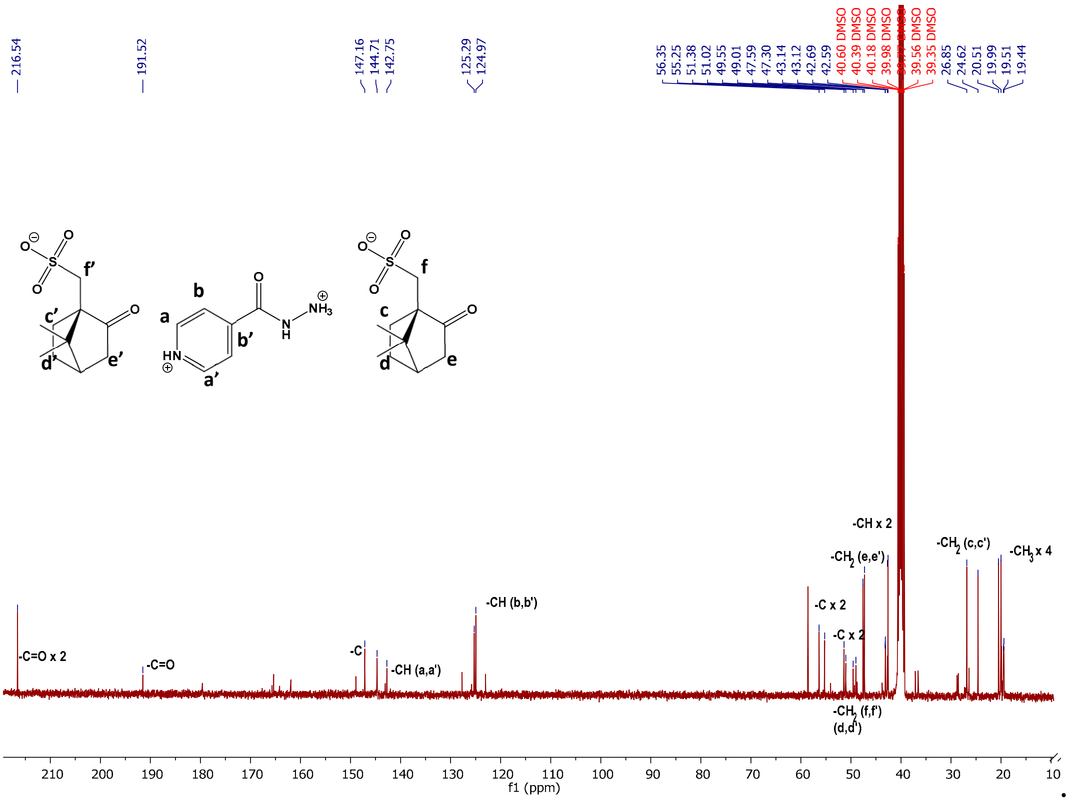

Figure S17. ^{13}C NMR spectra of [INH][R-CsO]$_2$.

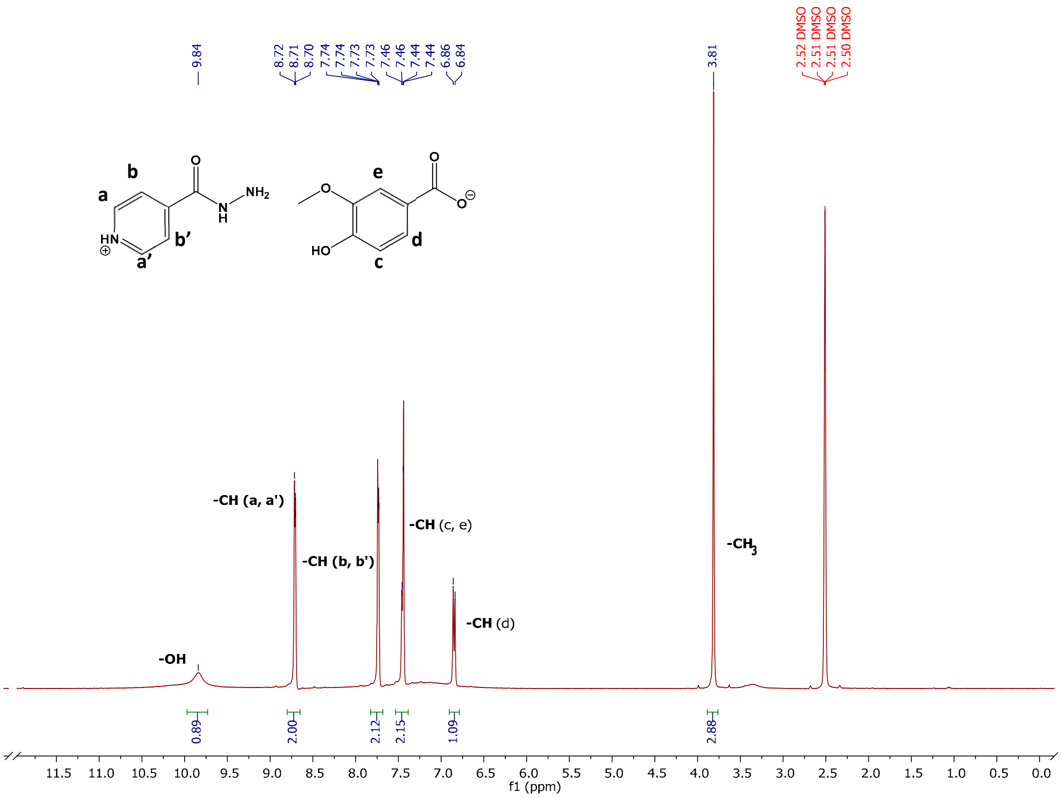

Figure S18. ^{1}H NMR spectra of [INH][VanO].

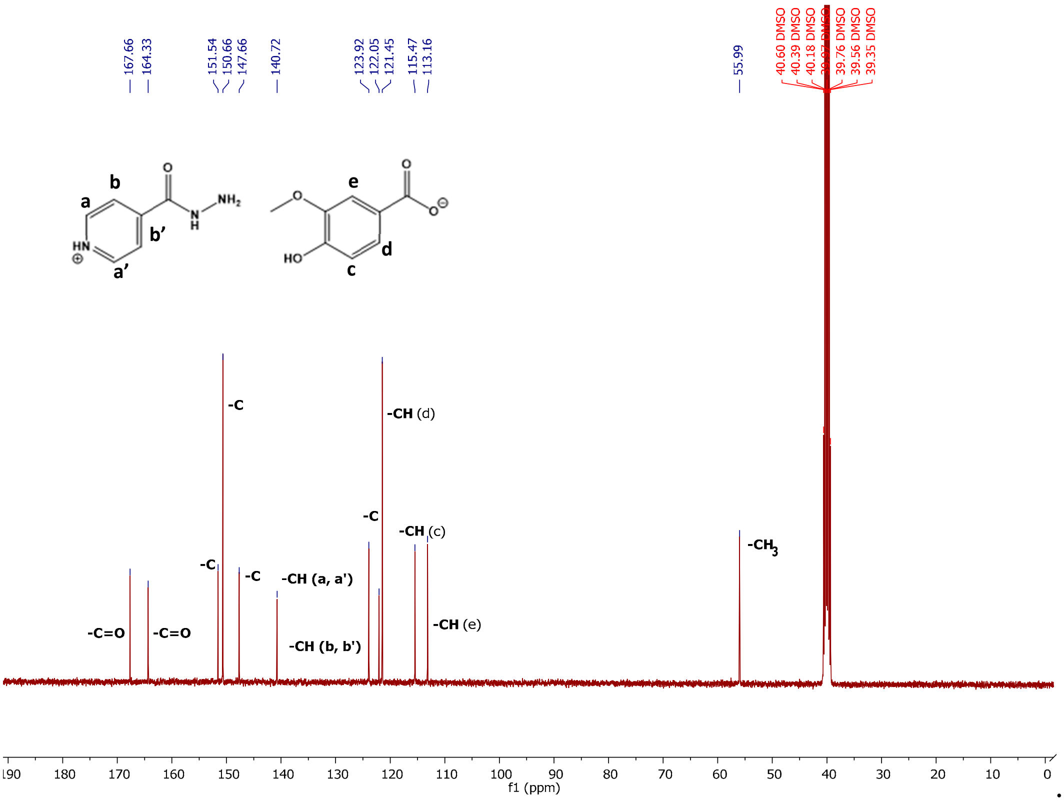

Figure s19. ^{13}C NMR spectra of [INH][VanO].

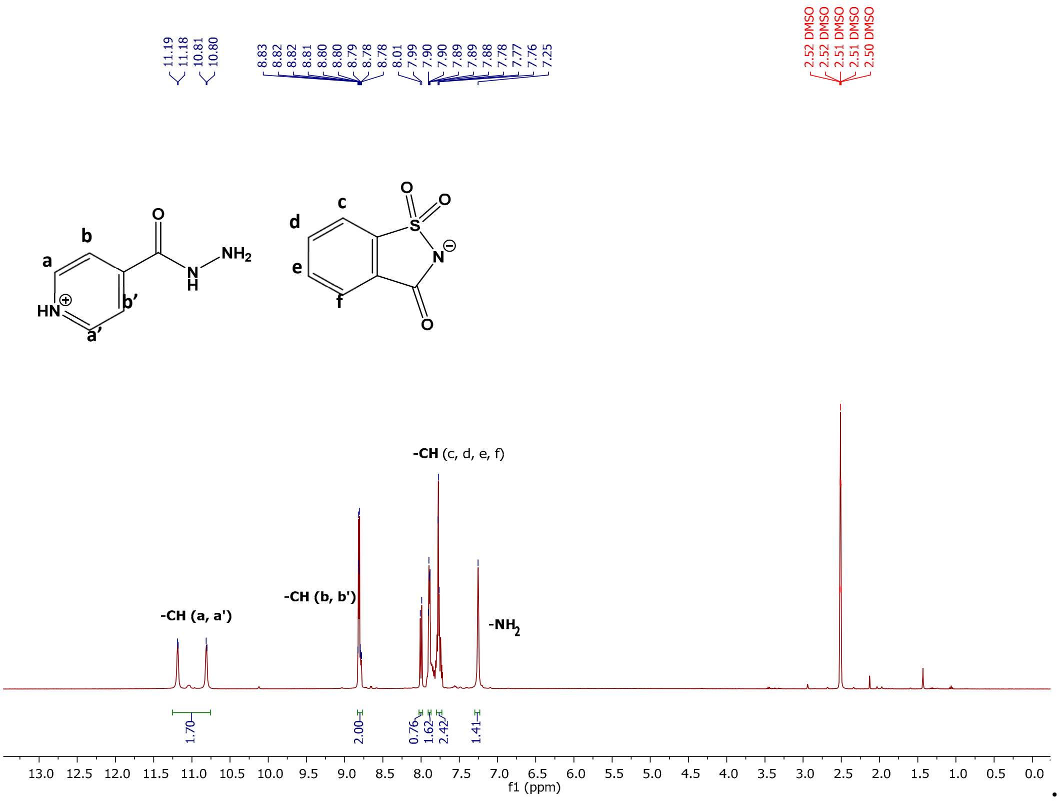

Figure S20. ^{1}H NMR spectra of [INH][Sac].

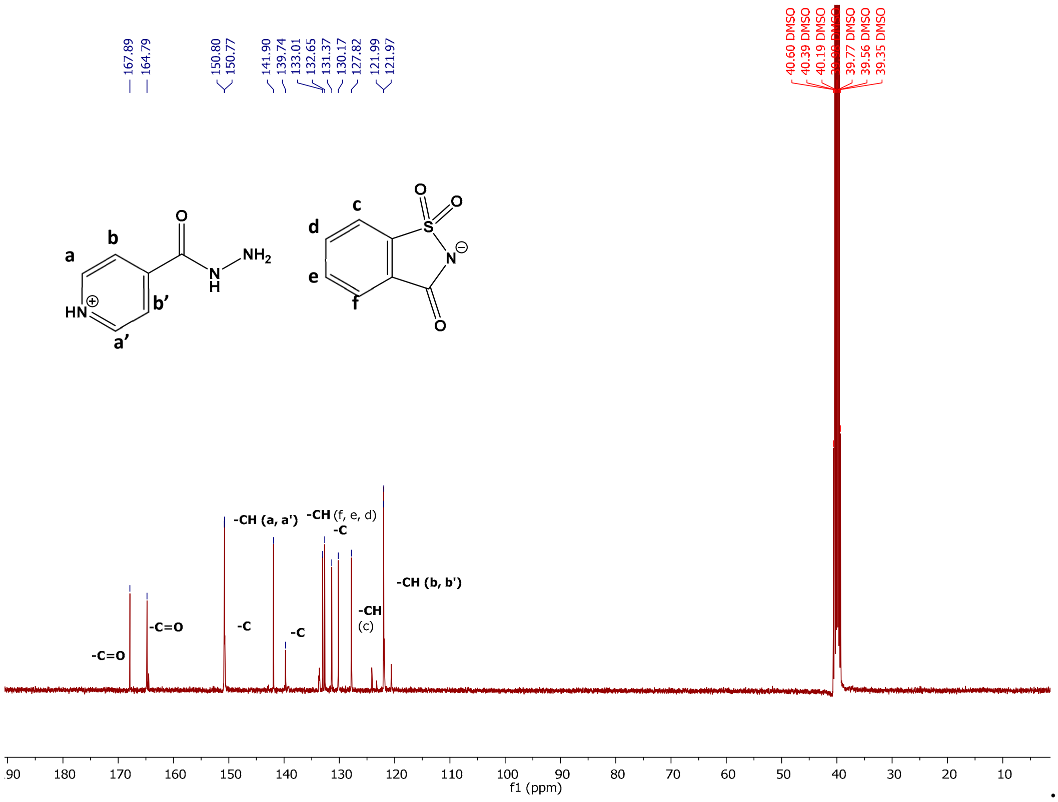

Figure S21. ^{13}C NMR spectra of [INH][Sac].

Appendix B@FTIR Spectra

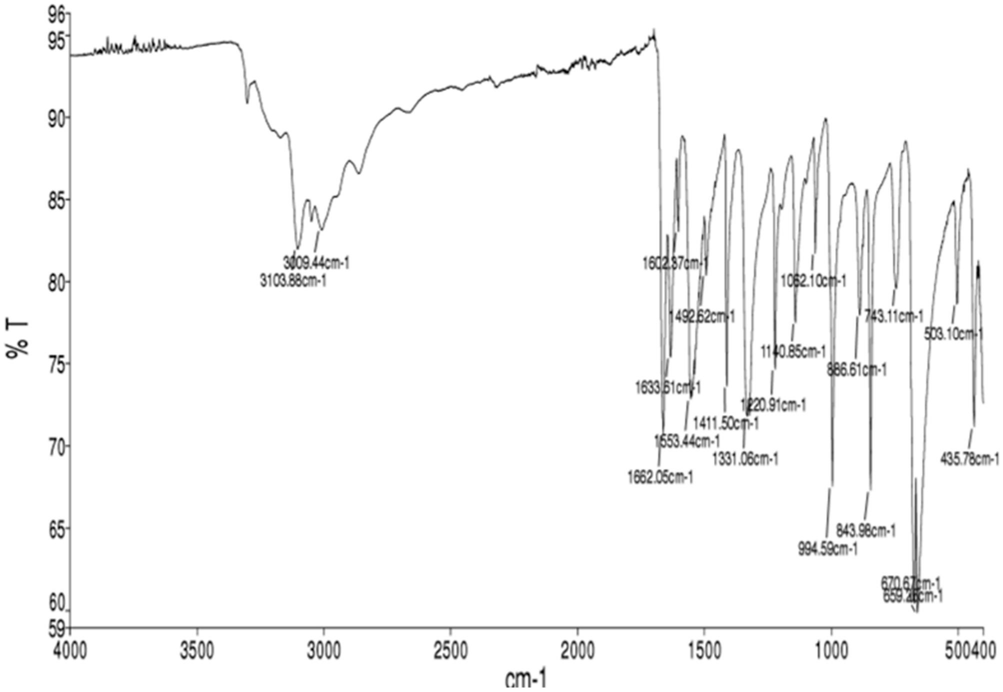

Figure S22. FTIR-ATR spectra of [INH].

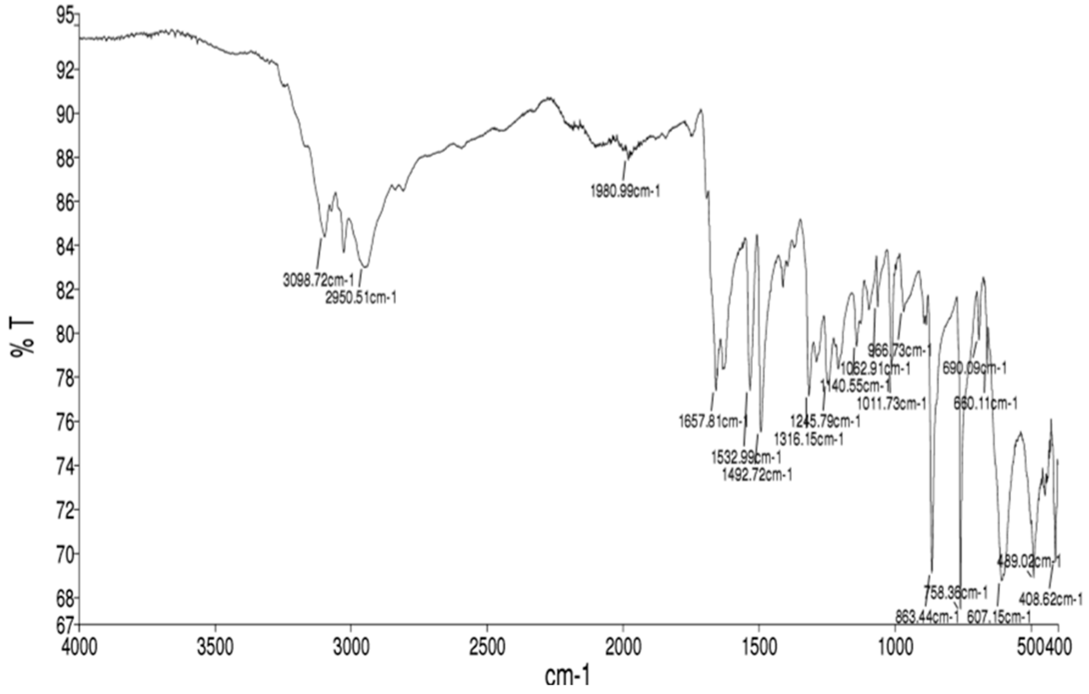

Figure S23. FTIR-ATR spectra of [INH][Cl].

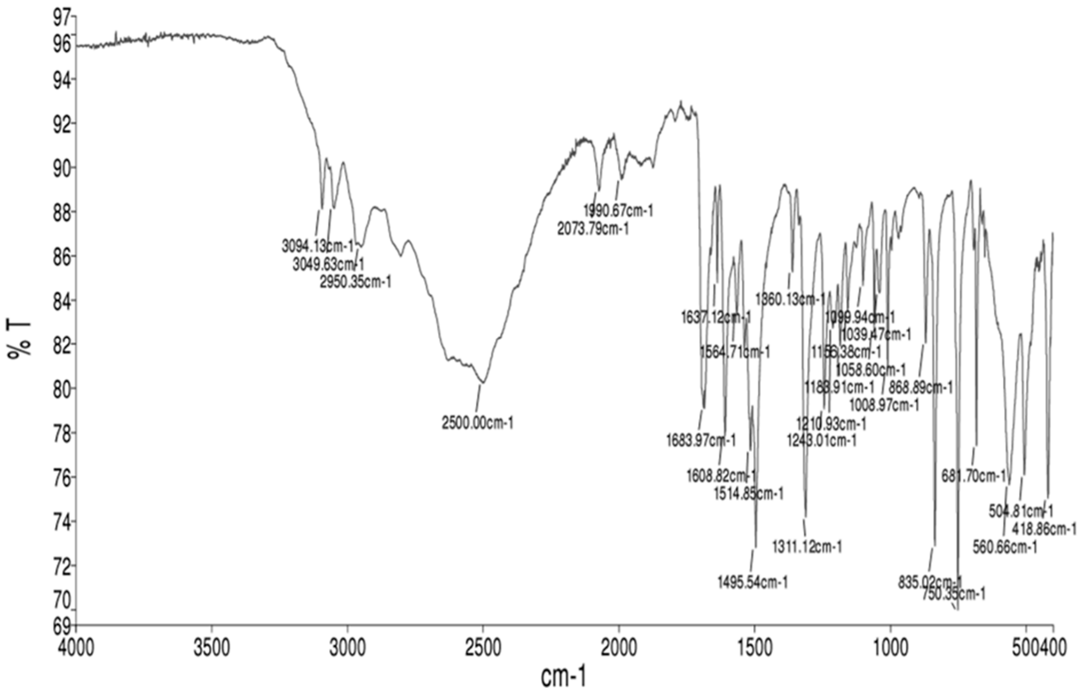

Figure S24. FTIR-ATR spectra of $[INH][Cl]_2$.

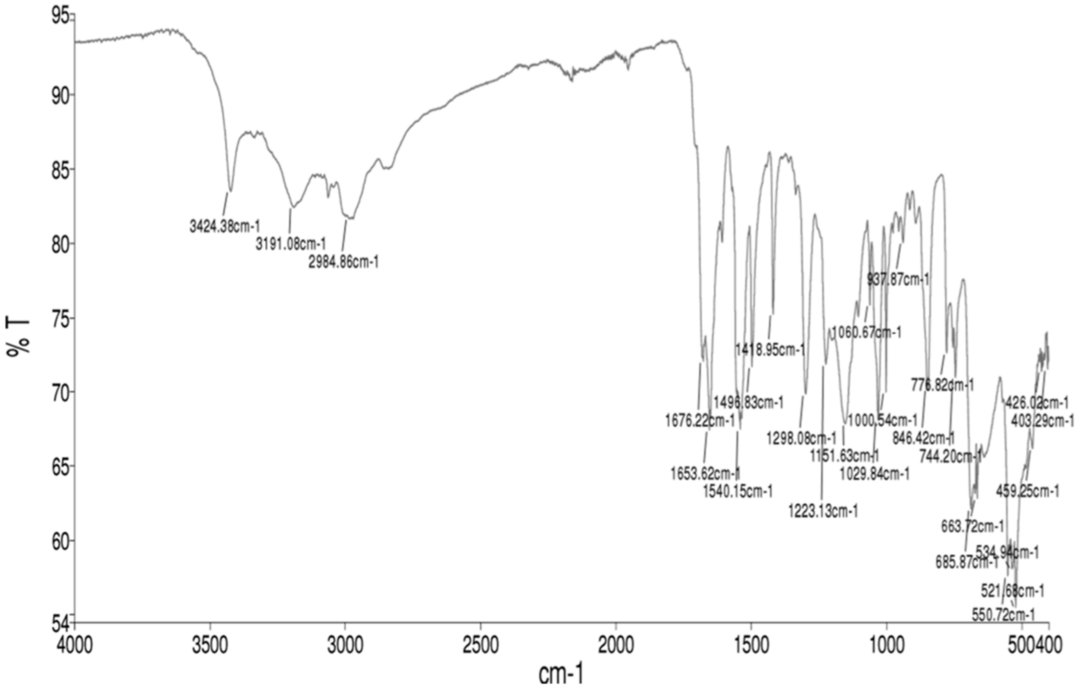

Figure S25. FTIR-ATR spectra of [INH][MsO].

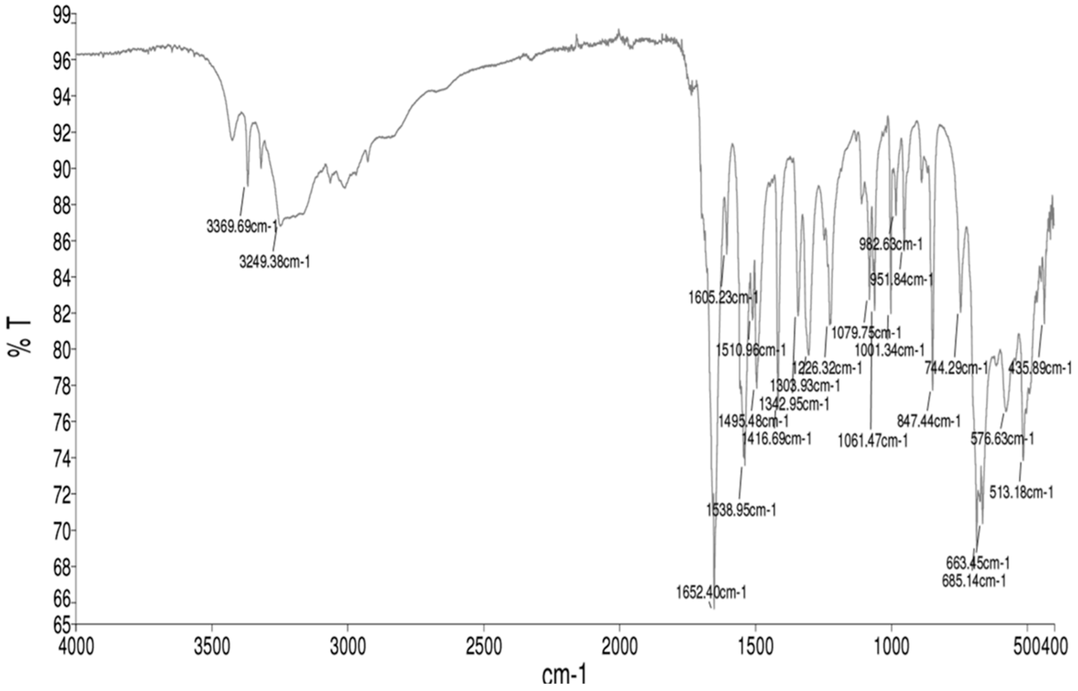

Figure S26. FTIR-ATR spectra of [INH][GcO].

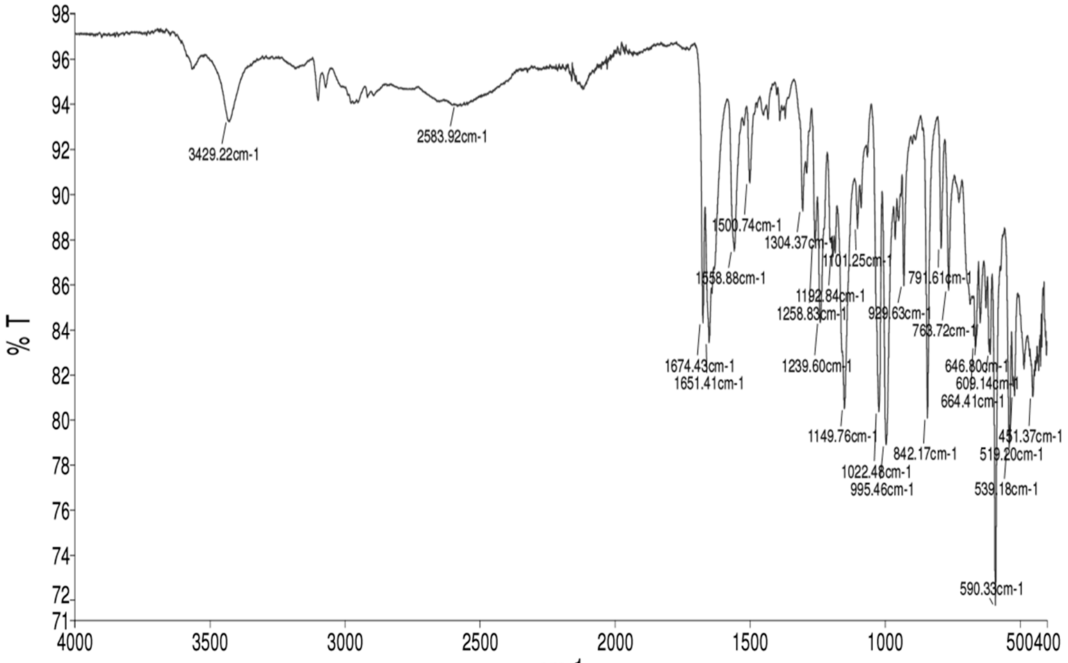

Figure S27. FTIR-ATR spectra of [INH][S-CsO].

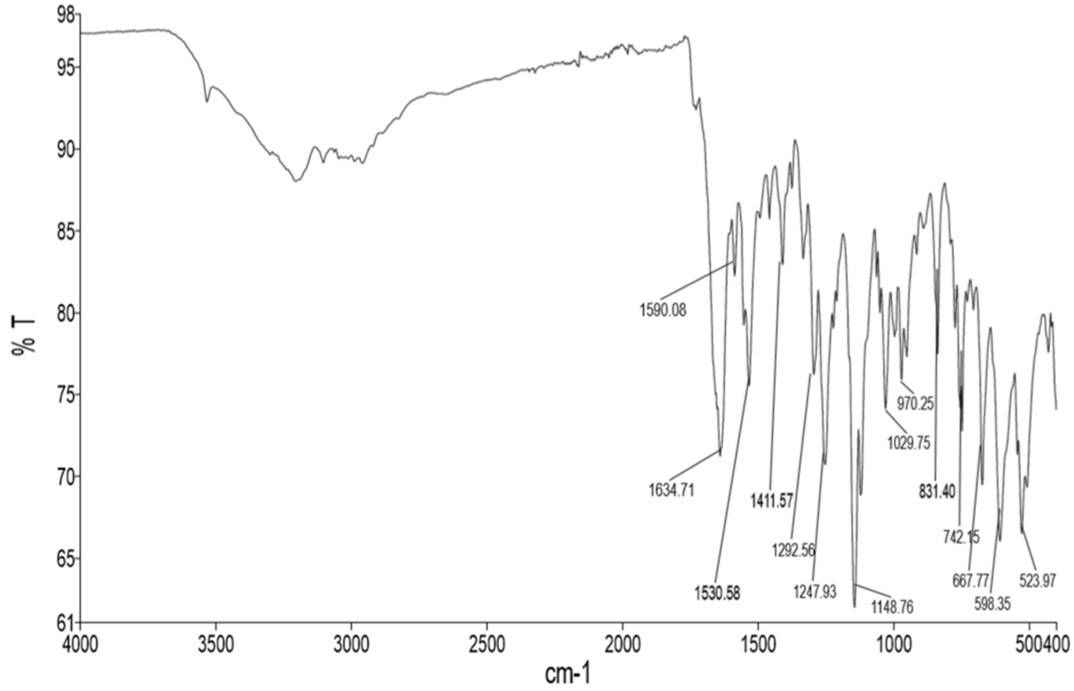

Figure S28. FTIR-ATR spectra of [INH][S-CsO]2.

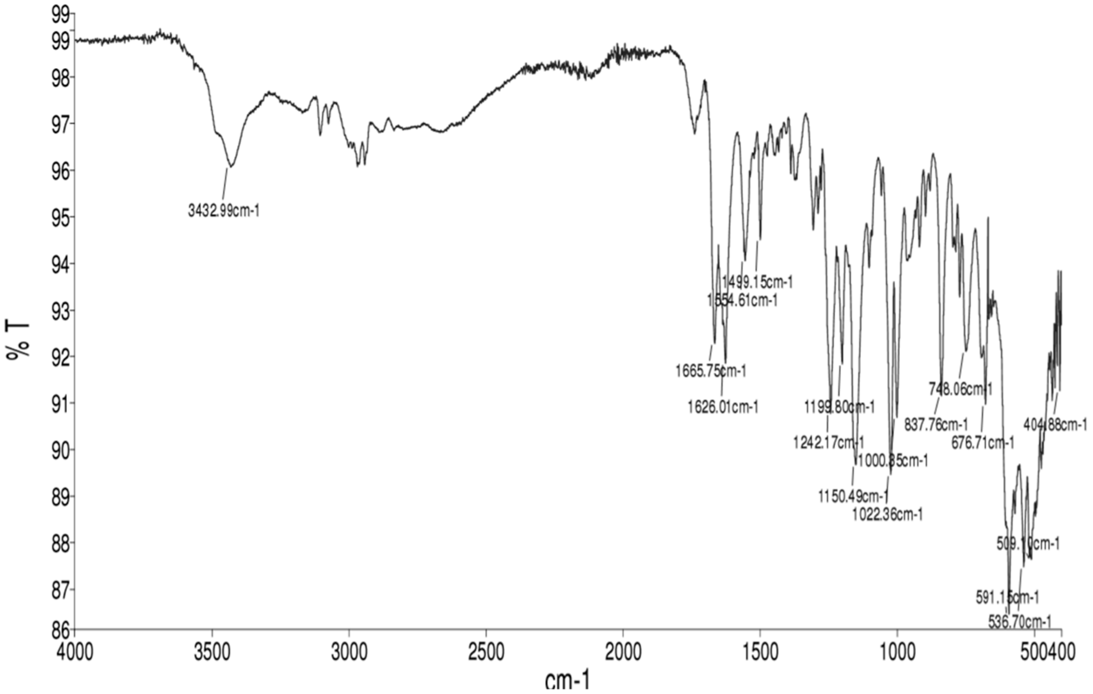

Figure S29. FTIR-ATR spectra of [INH][R-CsO].

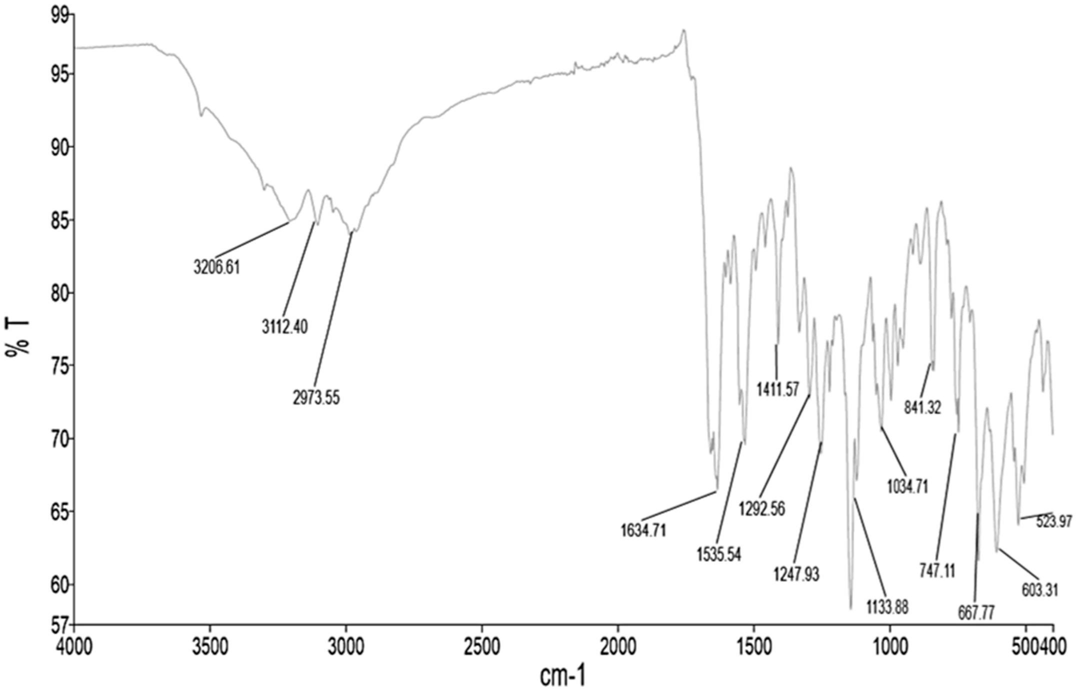

Figure S30. FTIR-ATR spectra of [INH][R-CsO]2.

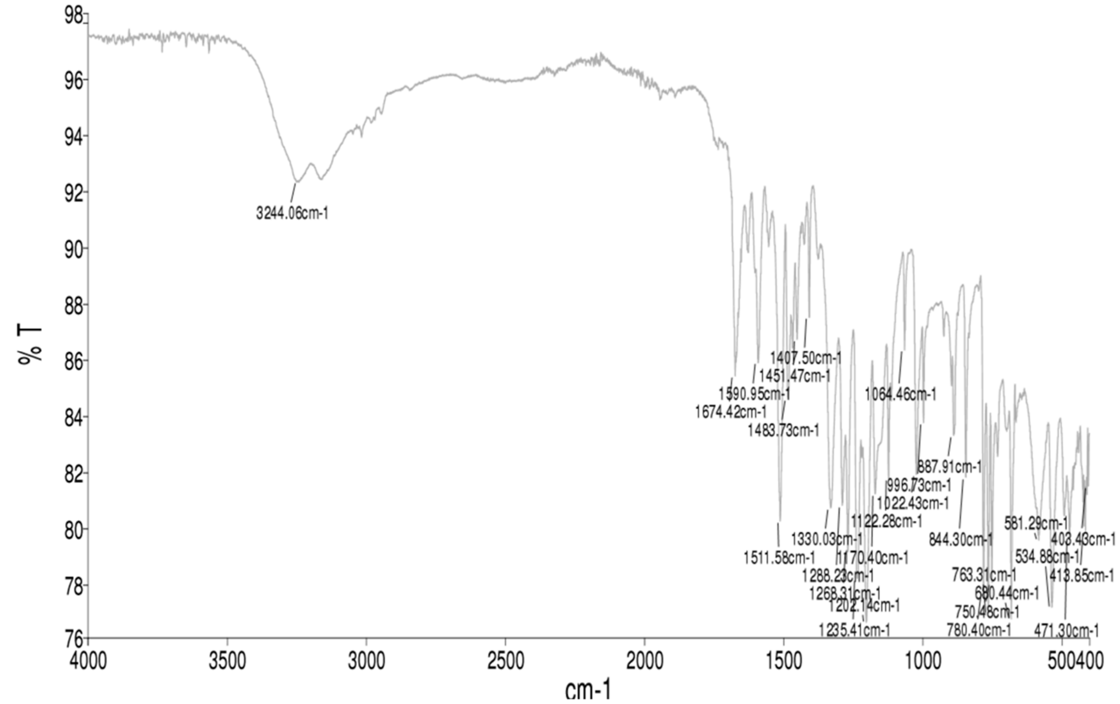

Figure S31. FTIR-ATR spectra of [INH][VanO].

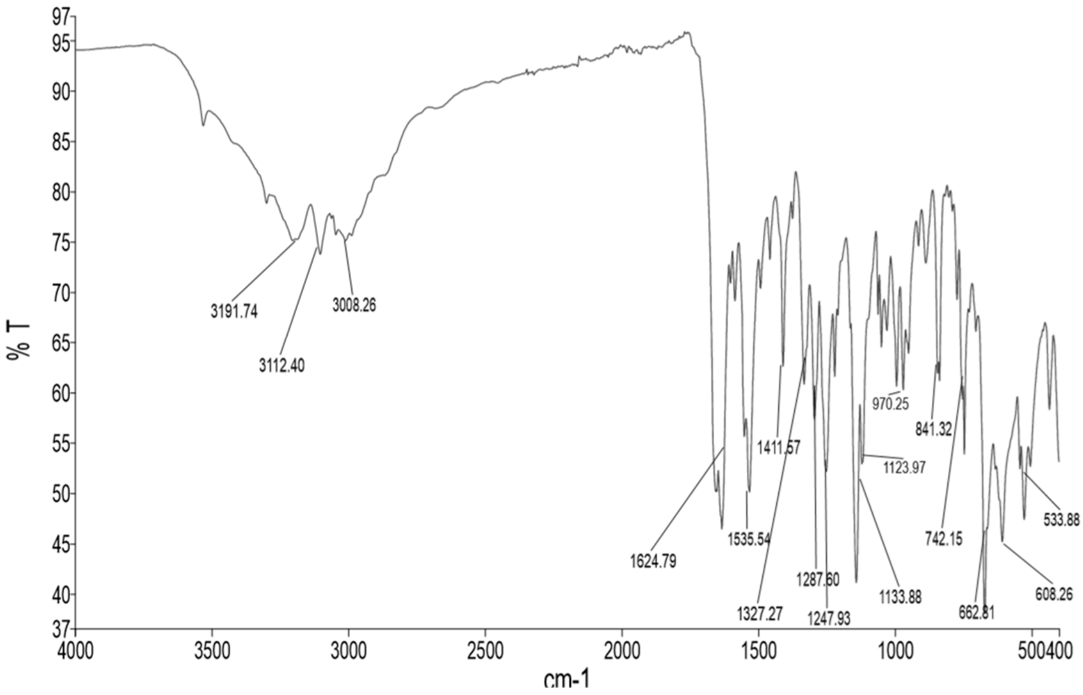

Figure S32. FTIR-ATR spectra of [INH][Sac].

Appendix C@DSC analysis

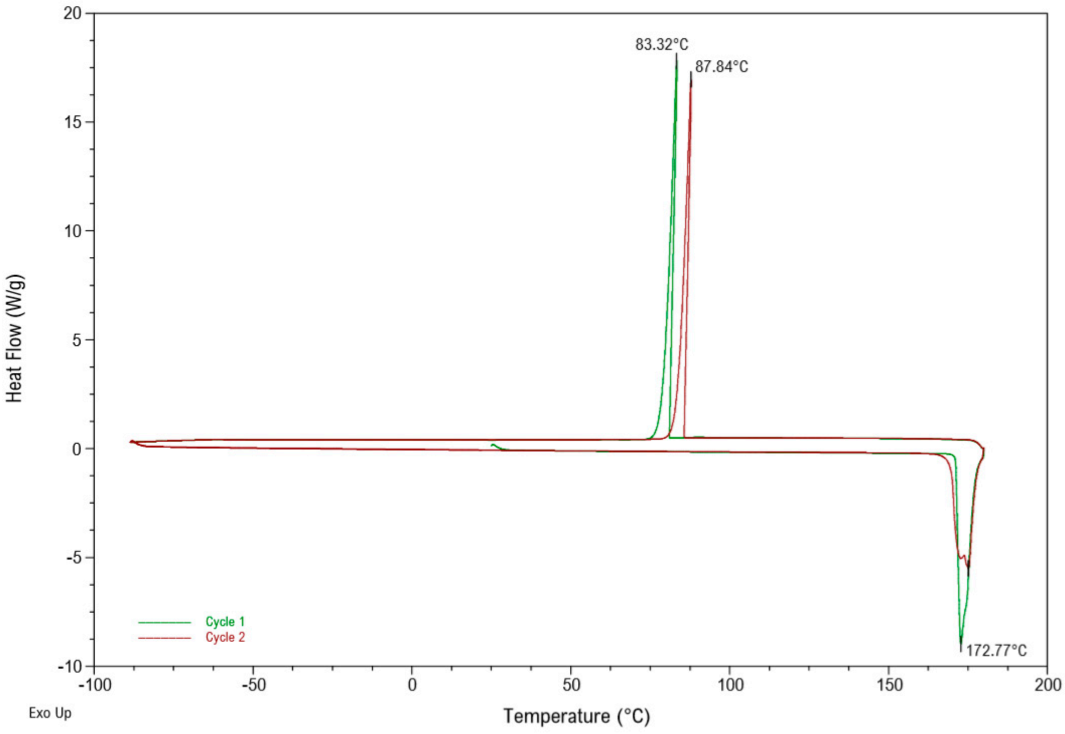

Figure S33. Thermogram of [INH] (DSC).

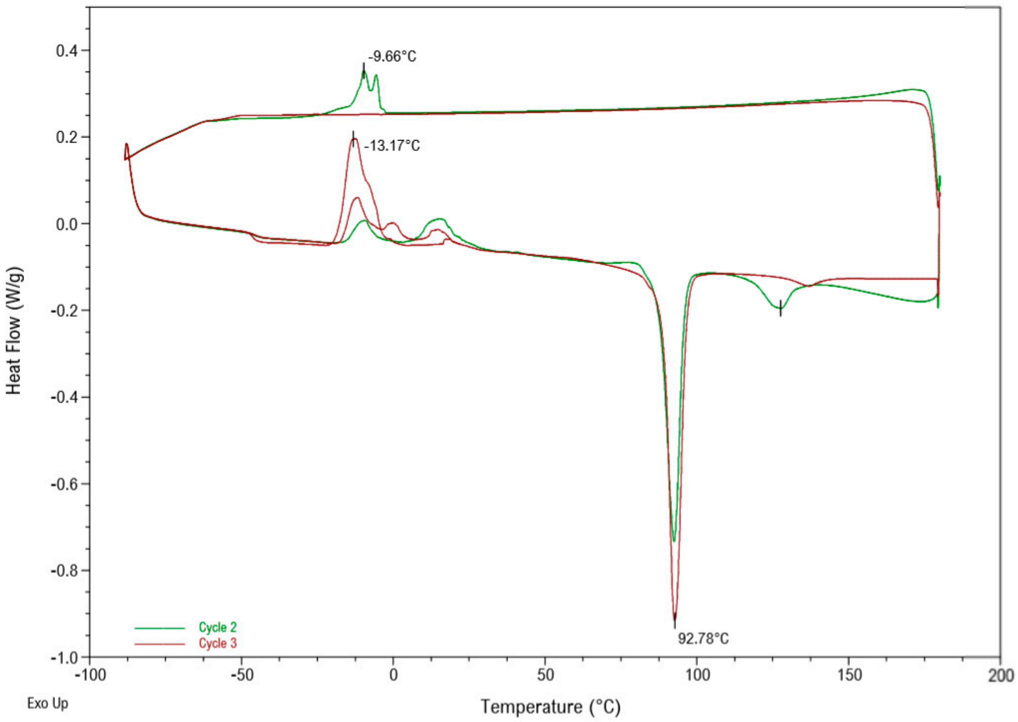

Figure S34. Thermogram of [INH][Cl] (DSC).

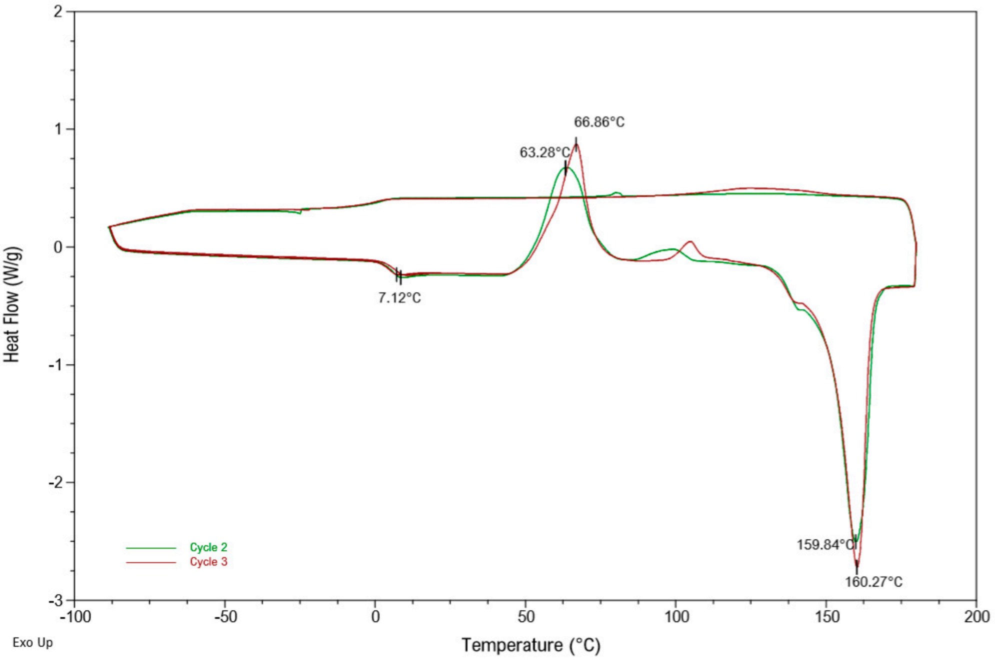

Figure S35. Thermogram of $[INH][Cl]_2$ (DSC).

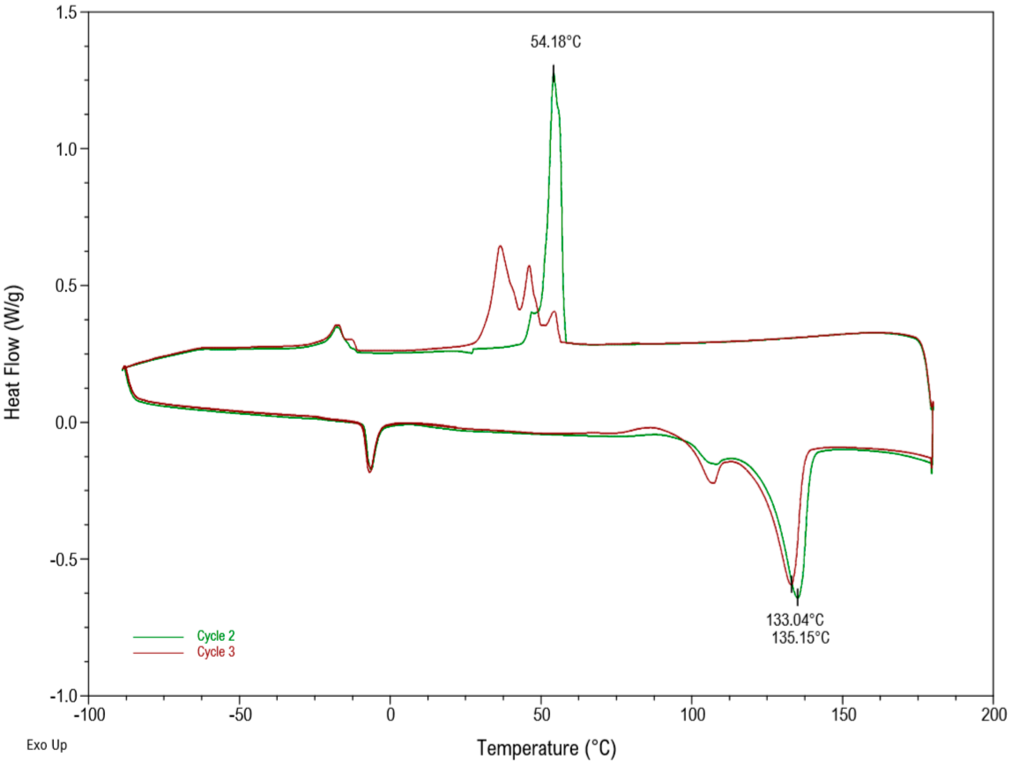

Figure S36. Thermogram of [INH][MsO] (DSC).

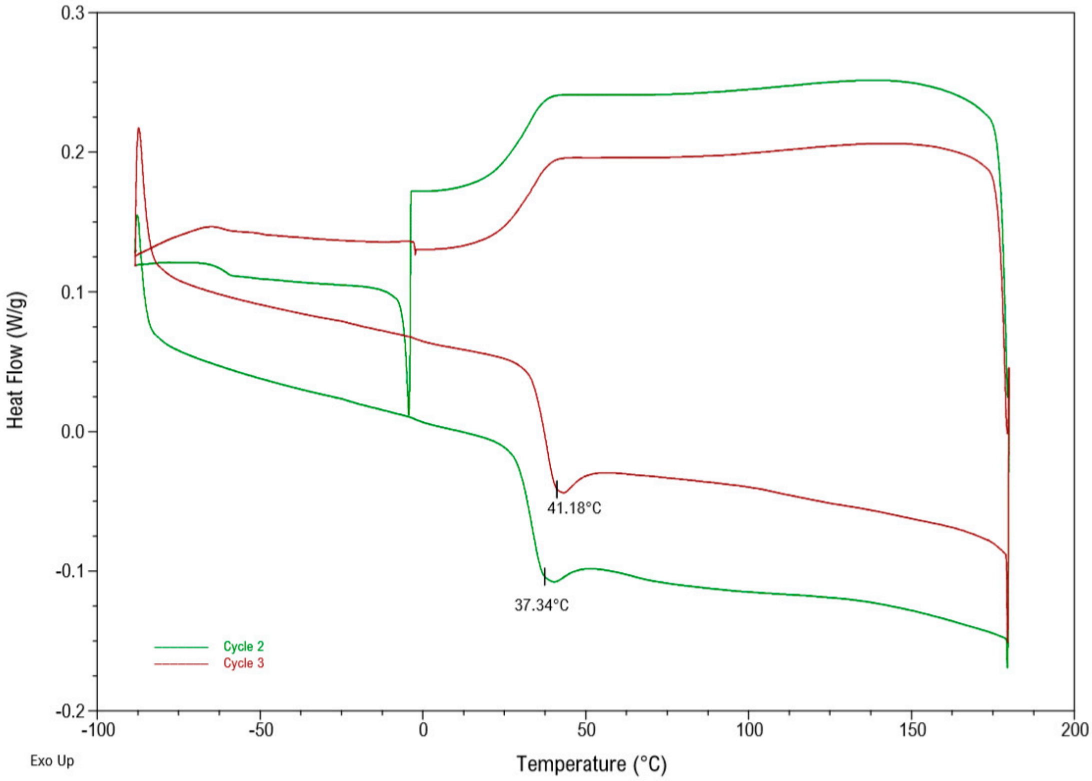

Figure S37. Thermogram of [INH][GcO] (DSC).

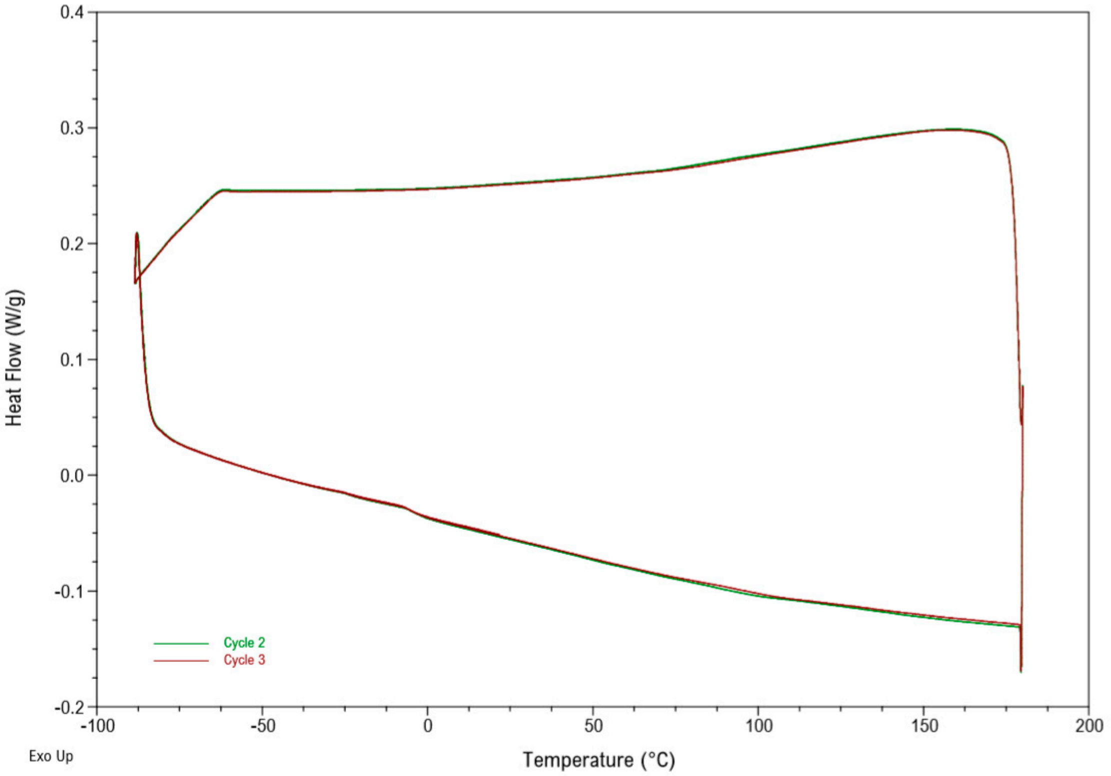

Figure S38. Thermogram of [INH][S-CsO] (DSC).

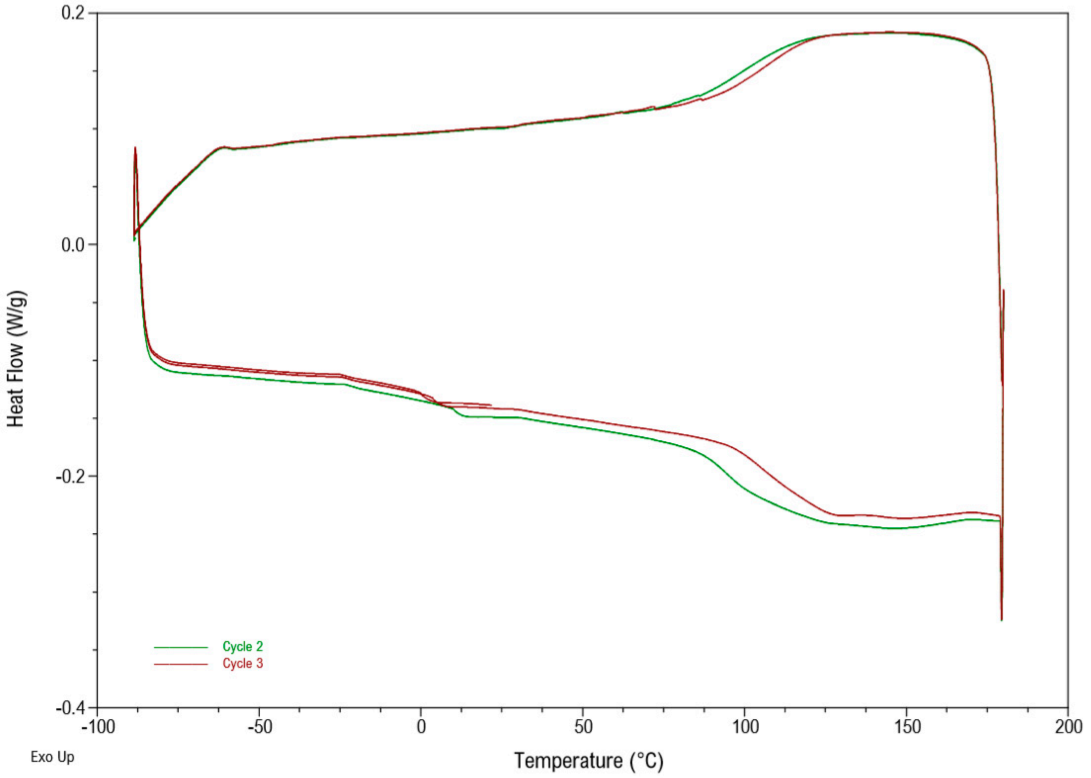

Figure S39. Thermogram of $[INH][S\text{-}CsO]_2$ (DSC).

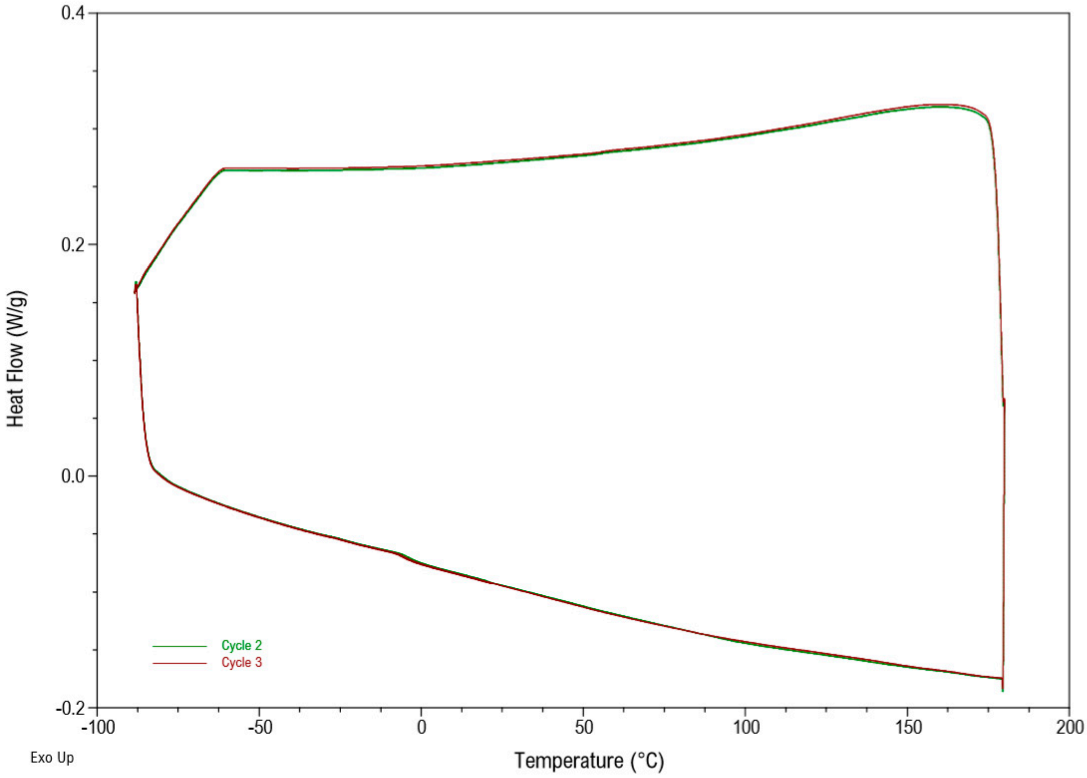

Figure S40. Thermogram of [INH][R-CsO] (DSC).

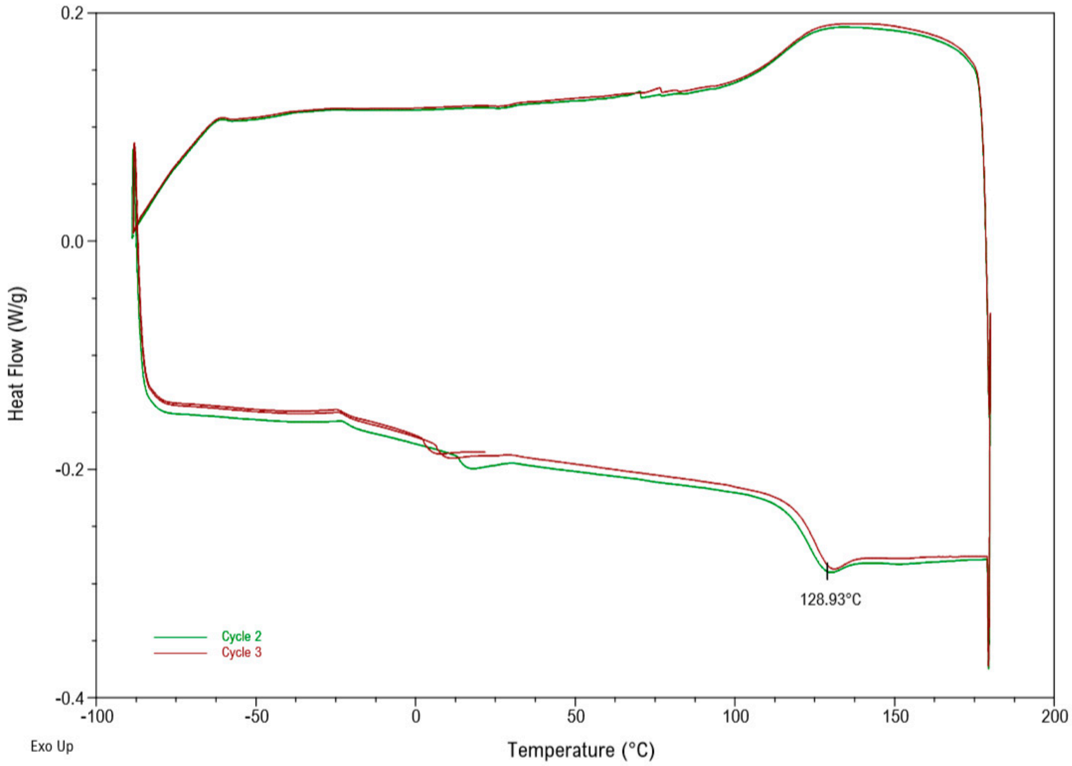

Figure S41. Thermogram of $[INH][R\text{-}CsO]_2$ (DSC).

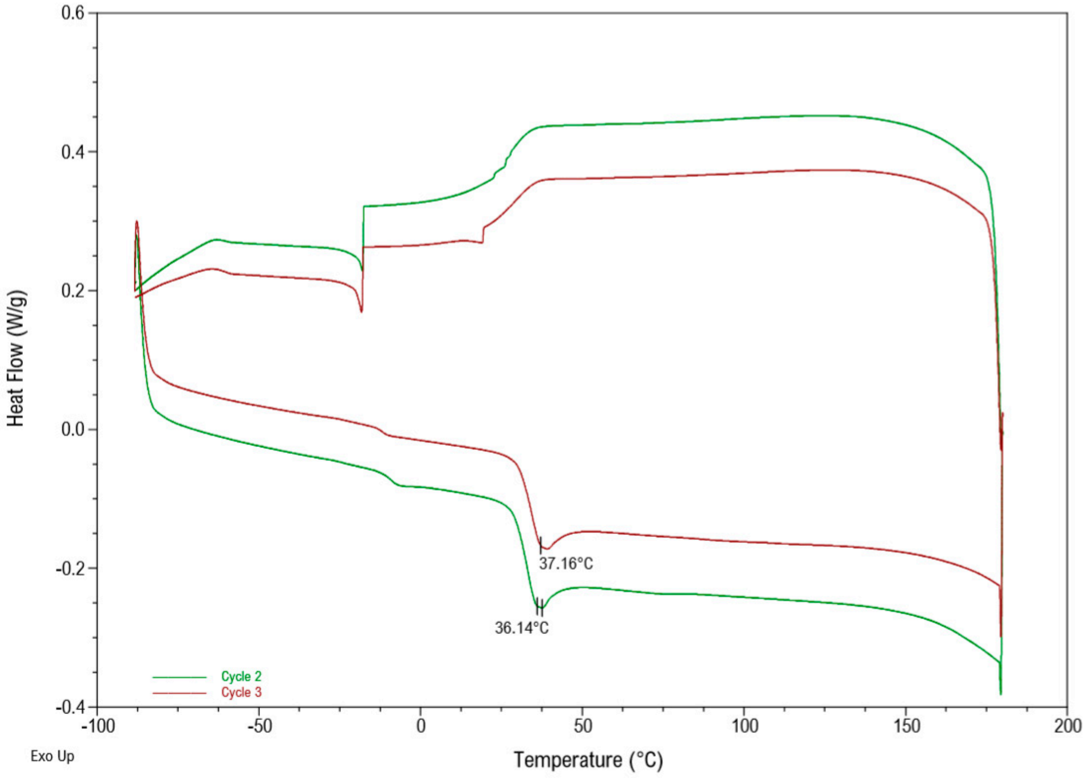

Figure S42. Thermogram of [INH][VanO] (DSC).

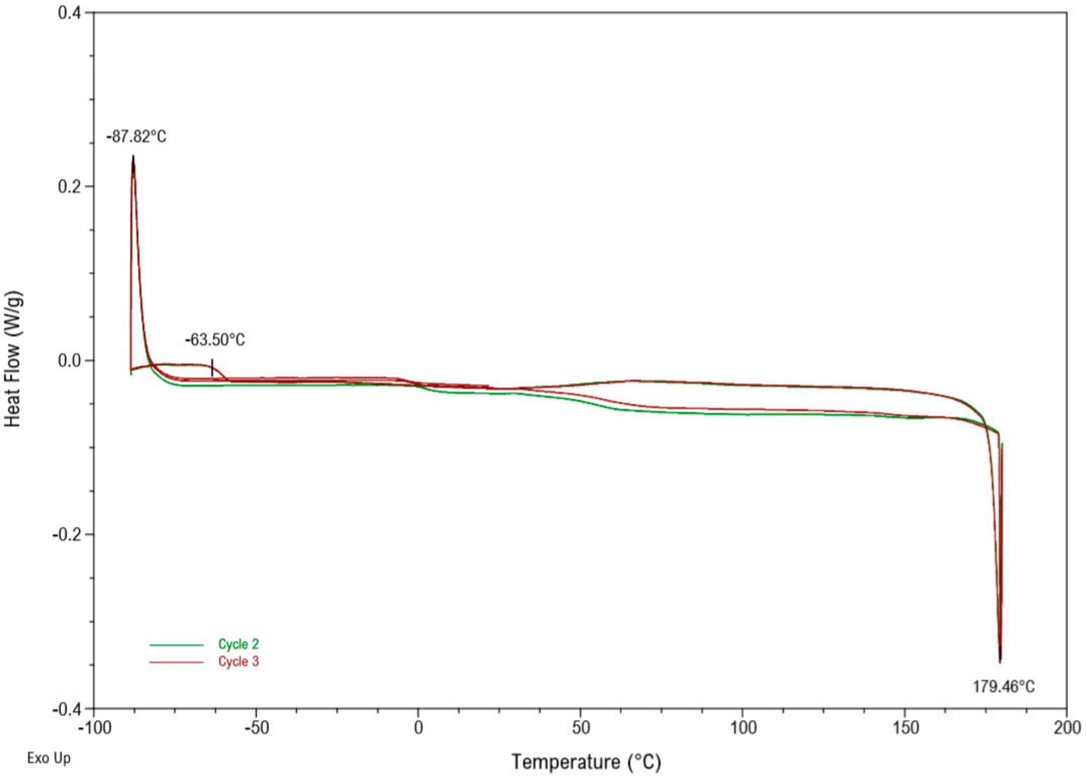

Figure S43. Thermogram of [INH][Sac] (DSC).
